# Somatic POLE exonuclease domain mutations elicit enhanced intratumoral immune responses in stage II colorectal cancer

**DOI:** 10.1136/jitc-2020-000881

**Published:** 2020-08-27

**Authors:** Shaobo Mo, Xiaoji Ma, Yaqi Li, Long Zhang, Ting Hou, Han Han-Zhang, Juanjuan Qian, Sanjun Cai, Dan Huang, Junjie Peng

**Affiliations:** 1 Department of Colorectal Surgery, Fudan University Shanghai Cancer Center, Shanghai, Shanghai, China; 2 Department of Oncology, Shanghai Medical College, Fudan University, Shanghai, Shanghai, China; 3 Department of Cancer Institute, Fudan University Shanghai Cancer Center, Shanghai, Shanghai, China; 4 Burning Rock Biotech, Guangdong, China; 5 Genecast Precision Medicine Technology Institute, Beijing, China; 6 Department of Pathology, Fudan University Shanghai Cancer Center, Shanghai, Shanghai, China

**Keywords:** biomarkers, tumor, cd8-positive t-lymphocytes, gastrointestinal neoplasms, tumor biomarkers

## Abstract

Previous studies found patients with POLE exonuclease domain mutations (EDMs) in targeted exons were related to significant better outcomes in stage II-III colorectal cancer (CRC). The detailed mutational profile of the entire POLE exonuclease domain, tumor mutation burden (TMB) and immune cell infiltration in POLE EDMs tumors, and the prognostic value of such mutations in stage II CRCs were largely unknown to us. This study was to clarify the characteristics, immune response and prognostic value of somatic POLE EDMs in stage II CRC. A total of 295 patients with stage II CRC were sequenced by next-generation sequencing with a targeted genetic panel. Simultaneous detection of the immune cells was conducted using a five-color immunohistochemical multiplex technique. The detailed molecular characteristics, tumor-infiltrating lymphocyte (TIL) and prognostic effect of POLE EDMs in stage II CRC were analyzed. For stage II CRCs, the POLE EDMs were detected in 3.1% of patients. Patients with POLE EDMs were more prone to be microsatellite instability-high (MSI-H) (33.3% vs 11.2%, p=0.043), younger at diagnosis (median 46 years vs 62 years, p<0.001) and more common at right-sided location (66.7% vs 23.1%; p=0.003). All patients with POLE EMDs were assessed as extremely high TMB, with a mean TMB of 200.8. Compared with other stage II CRCs, POLE EDMs displayed an enhanced intratumoral cytotoxic T cell response, evidenced by increased numbers of CD8+TILs and CD8A expression. Patients with stage II CRCs could be classified into three risk subsets, with significant different 5 years disease-free survival rates of 100% for POLE EDMs, 82.0% for MSI-H and 63.0% for MSS, p=0.013. In conclusion, characterized by a robust intratumoral T cell response, ultramutated POLE EDMs could be detected in a small subset of stage II CRCs with extremely high TMB. Patients with POLE EDMs had excellent outcomes in stage II CRCs, regardless of MSI status. Sequencing of all the exonuclease domain of POLE gene is recommended in clinical practice.

## Introduction

Colorectal cancer (CRC) is a disease of great heterogeneity. Compared with more advanced disease, stage II CRCs exhibit unique molecular heterogeneity, including chromosome instability, microsatellite instability (MSI), gene expression profiling and frequency of specific somatic mutations.^1–4^ Clinically, patients with stage II colon cancer have relatively good prognosis and the use of adjuvant chemotherapy has been controversial for all these patients.^5–8^ Within this group, additional clinicopathological variables, such as tumor/node/metastasis grade T4,^9 10^ perineural invasion,^11^ poorly differentiated histology,^12^ were studied to select the appropriate patients which would benefit the most from undergoing adjuvant chemotherapy, but the results were still unsatisfactory.^13^

With the development of precision medicine, more biomarkers were identified and applied for risk stratification and patient selection in CRCs.^14^ In stage II colon cancer, MSI and mismatch repair deficiency (dMMR) are the most important biomarkers and are widely used to help clinicians choose adjuvant treatment and predict patients’ outcomes.^15^ However, approximately 85% of patients with stage II colon cancers are classified as microsatellite stable (MSS) or proficient MMR (pMMR),^16^ and the biomarkers lack for these patients. Several gene expression-based biomarkers,^17–19^ such as Oncotype DX, were reported to improve outcome prediction or adjuvant treatment selections.^18^

Recently, through large-scale next-generation sequencing (NGS), the Cancer Genome Atlas (TCGA) Research Network reported numerous genomic aberrations associated with the development or progression of CRC,^20^ and this study was considered an essential first step toward precision medicine in CRCs. Also, using Memorial Sloan Kettering Cancer Center (MSK)-IMPACT, a capture-based NGS platform that can detect mutations and copy-number alterations, and select rearrangements in 341 or more cancer genes, MSK cohort provided a resource for further studies of the biology of CRC.^21^ Similar to other tumors, in addition to the well-established driver genes, low-frequency mutations or other oncogenic events were also discovered by NGS test.^20 21^ The DNA polymerase genes ɛ (POLE) and δ (POLD1) were two potential biomarkers in CRC of low mutation frequencies. While germline POLD1 mutations might be related to familial cancers,^22^ the oncogenic value of somatic POLD1 exonuclease domain mutations (EDMs, also referred to proofreading domain mutations) is still controversial. POLE mutations of proofreading exonuclease domain were reported in approximately 1%–3% of CRC^20 23^ and 5%–7% of endometrial cancers (ECs).^24^ Previous studies found patients with POLE proofreading EDMs in targeted exons were related to significant better outcomes in stage II–III CRCs.^25^ In ECs, ultramutated POLE EDMs ECs with excellent prognosis, are characterized by a robust intratumoral T cell response, which correlates with, and may be caused by an enrichment of antigenic neopeptides.^26^ However, the detailed mutational profile of the entire POLE exonuclease domain, immune cell infiltration in POLE EDMs tumors and the prognostic value of such mutations in stage II CRCs were largely unknown to us.

Therefore, in current study, the published TCGA and MSKCC series were included to analyze the mutational profile and immune response of POLE EDMs in stage II CRCs. We also conducted a genetic mutation assay by NGS in a retrospective series of stage II CRCs, in order to further clarify the prognostic value of POLE EDMs. A comprehensive investigation into immune cell infiltration provides more accurate and reliable evidence of increased immunogenicity for stage II CRC patients with POLE EDMs. Two panels were, thus, developed to simultaneous detection of the immune constituents CD3+, CD8+, CD45RO+, PD-1+, PD-L1+ (Panel 1) and CD4+, FOXP3+, CD68+, CD163+, PD-L1+ (Panel 2) cells in stage II CRCs using a five-color immunohistochemical multiplex technique.

## Results

### Patient Characteristics and outcomes

A total of 295 patients with stage II CRC were retrospectively collected from the Fudan University Shanghai Cancer Center (FUSCC) database. Also, 183 eligible patients from TCGA dataset and 133 cases from MSKCC cohort were included in current study. The clinicopathological characteristics and treatment information according to stage II CRC POLE status were listed in table 1 (see also online supplementary figure 1, online supplementary tables 1 and 2).

10.1136/jitc-2020-000881.supp1 Supplementary data

10.1136/jitc-2020-000881.supp8 Supplementary data

10.1136/jitc-2020-000881.supp9 Supplementary data

**Table 1:** Demographic and clinicopathological characteristics according to stage II CRC POLE status in FUSCC cohort

Characteristic	POLE EDMs	POLE wild-type and POLE non-EDMs	P value
Total	9 (3.1)	286 (96.9)	--
Age (IQR)	46 (43–54)	62 (54–71)	<0.001
Gender			0.162
Female	2 (22.2)	131 (45.8)	
Male	7 (77.8)	155 (54.2)	
Histological type			0.196
Adenocarcinoma	6 (66.7)	238 (83.2)	
Mucinous adenocarcinoma	3 (33.3)	48 (16.8)	
Pathological grade			0.372
Well	0 (0)	18 (6.3)	
Moderate	5 (55.6)	200 (69.9)	
Poor	3 (33.3)	59 (20.6)	
Unknown	1 (11.1)	9 (3.2)	
Location			0.003
Right	6 (66.7)	66 (23.1)	
Left	3 (33.3)	220 (76.9)	
pT stage			0.832
T3	5 (55.6)	169 (59.1)	
T4	4 (44.4)	117 (40.9)	
LNH			0.498
<12	1 (11.1)	58 (20.3)	
≥12	8 (88.9)	228 (79.7)	
Lymphovascular invasion			0.003
Negative	4 (44.4)	237 (82.9)	
Positive	5 (55.6)	49 (17.1)	
Perineural invasion			0.264
Negative	8 (88.9)	206 (72.0)	
Positive	1 (11.1)	80 (28.0)	
Pretreatment CEA			0.541
Negative	8 (88.9)	231 (80.8)	
Positive	1 (11.1)	55 (19.2)	
Chemotherapy			0.663
No	3 (33.3)	116 (40.6)	
Yes	6 (66.7)	170 (59.4)	
MSI status			0.043
MSS	6 (66.7)	254 (88.8)	
MSI-H	3 (33.3)	32 (11.2)	
RAS			0.109
Wild-type	2 (22.2)	141 (49.3)	
Mutation	7 (77.8)	145 (50.7)	
BRAF			0.116
Wild-type	7 (77.8)	264 (92.3)	
Mutation	2 (22.2)	22 (7.7)	
PTEN			<0.001
Wild-type	3 (33.3)	265 (92.7)	
Mutation	6 (66.7)	21 (7.3)	
PIK3CA			<0.001
Wild-type	1 (11.1)	227 (79.4)	
Mutation	8 (88.9)	59 (20.6)	

CEA, carcinoembryonic antigen; CRC, colorectal cancer; EDM, exonuclease domain mutation; FUSCC, Fudan University Shanghai Cancer Center; LNH, lymph nodes harvested; MSI-H, microsatellite instability-high; MSS, microsatellite stabilization.

In FUSCC database, 95 patients were recurred, while the other 200 patients were still free of disease at the last follow-up, with a median follow-up time of 60.4 months (ranging from 7 to 95 months). A total of 59.7% of patients underwent adjuvant chemotherapy with 5-fluorouracil (5-Fu)-based monotherapy or combined therapy. The 5 years disease-free survival (DFS) rate was 66.7%, and the 5 years overall survival (OS) rate was 82.6% for all patients.

### POLE mutations, tumor mutation burden and MSI status in stage II CRC

Of the 295 patients in FUSCC dataset, 35 patients (11.9%) were classified MSI-H by NGS. All somatic mutations of POLE were detected in 11.5% (34 cases) of patients. However, the POLE EDMs were only detected in 3.1% of patients (nine cases). Compared with POLE wild-type or non-EDMs patients, patients with POLE EDMs were younger at diagnosis (median 46 years vs 62 years, p<0.001), had tumors localized more commonly in right-sided colons (66.7% vs 23.1%, p=0.003), had higher lymphovascular invasion (55.6% vs 17.1%, p=0.003) and higher frequency of MSI-H status (33.3% vs 11.2%, p=0.043), PTEN mutation (66.7% vs 7.3%, p<0.001) and PIK3CA mutation (88.9% vs 20.6%, p<0.001). The detailed information was listed in table 1. Also, the POLE EDMs were detected in 3.8% of patients (seven cases) and 3.0% of patients (four cases) in TCGA and MSKCC cohorts, respectively (online supplementary figure 1A). Besides, POLE EDMs patients were younger at diagnosis (median 59 years vs 69 years, p=0.049) and had higher frequency of pT4 status (28.6% vs 6.2%, p=0.024) in TCGA cohort (online supplementary table 1). Furthermore, in TCGA and MSKCC cohorts, based on POLE EDMs and MSI status, we classified all patients with stage II CRCs into three groups: POLE EDMs (regardless of MSI status), MSI-H and MSS. As demonstrated in online supplementary figure 1C and online supplementary figure 2A, molecular subtype of POLE EDMs had the highest frequency of somatic mutations, compared with both MSI-H and MSS subtypes. The entire mutation spots of POLE on protein structure were displayed in lollipop plots (online supplementary figure 1D and online supplementary figure 2B), including a range of mutation types.

10.1136/jitc-2020-000881.supp2 Supplementary data

For each POLE EDMs, NGS-assessed MSI status, detailed mutation sites, immunohistochemistry (IHC)-tested MMR status and tumor mutation burden (TMB) were listed in online supplementary table 3 and online supplementary table 4. Hotspot mutations in exon 9 (P286R), 13 (V411L) and 14 (S459F) were detected in two cases, one case, one case in FUSCC cohort, and two cases, three cases, zero case in TCGA cohort, and two cases, zero case, one case in MSKCC cohort. Besides, two concurrent mutations at exonuclease domain were detected in one case in both FUSCC cohort (R413M and A448T) and MSKCC cohort (D275G and S459F). The detected mutations varied at different exons. In FUSCC cohort, by NGS-assessed MSI status, six patients (66.7%) with POLE EDMs were classified as MSS, and the other three patients (33.3%) were MSI-H (online supplementary figure 3). However, of the three patients classified as MSI-H, only one patient was classified as dMMR by IHC (loss of expression in hMLH1 and PMS2). For this patient, further studies confirmed that there was no somatic mutation in MMR genes and the dMMR status was caused by hMLH1 methylation (online supplementary figure 4).

10.1136/jitc-2020-000881.supp10 Supplementary data

10.1136/jitc-2020-000881.supp11 Supplementary data

10.1136/jitc-2020-000881.supp3 Supplementary data

10.1136/jitc-2020-000881.supp4 Supplementary data

In FUSCC dataset, for all patients with POLE EDMs, we further tested TMB by a larger gene panel with 520 cancer related genes. All the nine patients were classified as high TMB; the mean TMB for the nine patients with POLE EDMs were 200.8 per Mb (ranging from 54 to 499.2 per Mb). Also, the mutation counts were extremely high in POLE EDMs in both TCGA and MSKCC cohorts (online supplementary figure 5).

10.1136/jitc-2020-000881.supp5 Supplementary data

### Immune response according to POLE EDMs and MMR status

**Figure 1:**
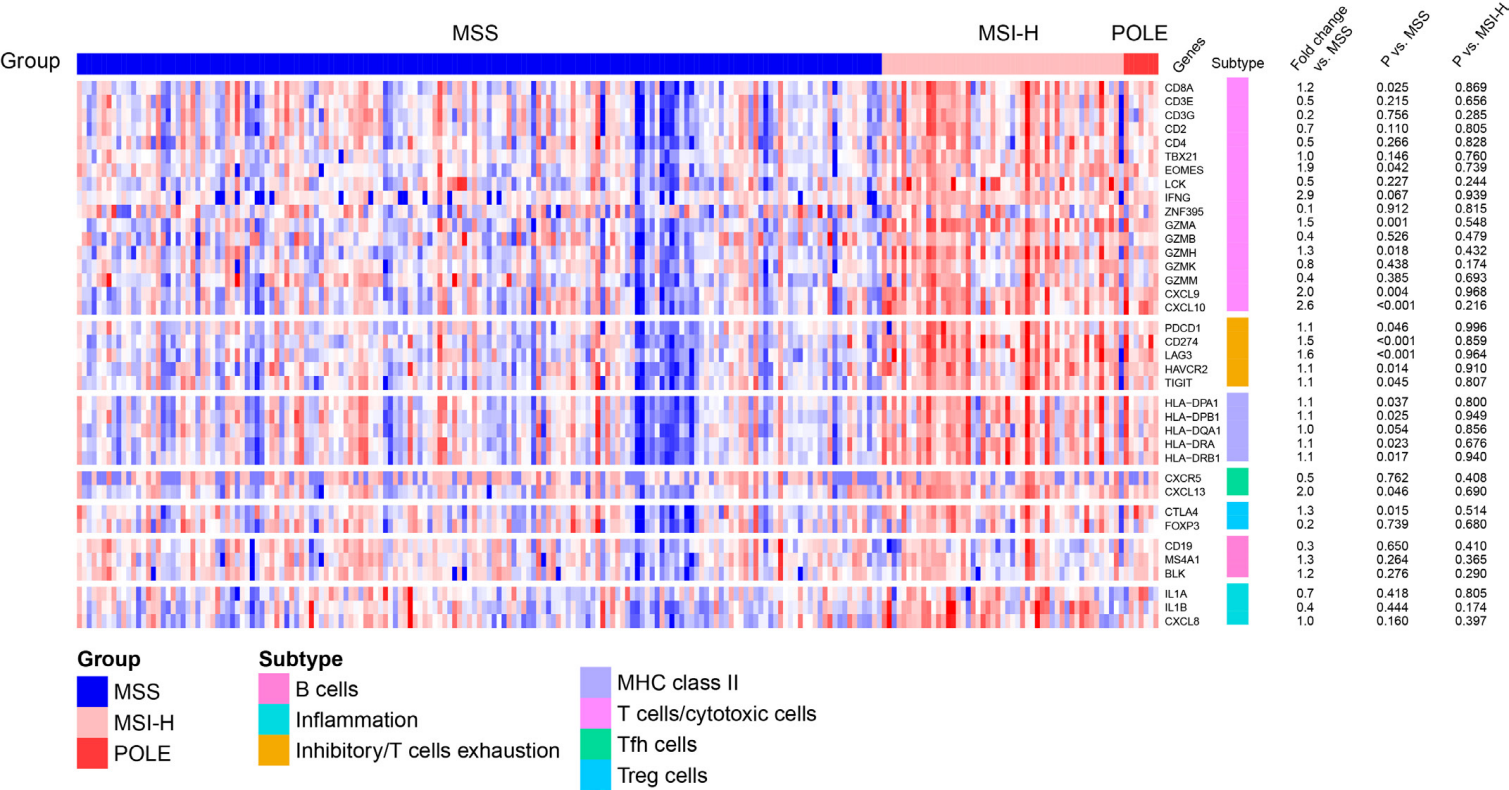
T cell response according to tumor molecular subtype of stage II CRCs in TCGA. Heatmap showing expression of immunological genes according to pole EDMs, MSI-H and MSS three subgroups. CRCs, colorectal cancer; EDMs, exonuclease domain mutations; MSI-H, microsatellite instability-high; MSS, microsatellite stable; TCGA, the Cancer Genome Atlas.

In TCGA dataset, a heat map of differentially expressed immune related genes (figure 1) demonstrated that POLE EDMs tumors in stage II CRCs have high expression of a large set of immune-related genes compared with MSS cancers. Focused analysis of genes involved in T cell-mediated cytotoxicity confirmed that, compared with MSS tumors, POLE EDMs demonstrated upregulation of CD8A (1.2 fold vs MSS stage II CRCs, p=0.025), accompanied by significant increases in EOMES (1.9-fold, p=0.042), GZMA (1.5-fold, p=0.001), GZMH (1.3-fold, p=0.018), CXCL9 (twofold, p=0.004), and CXCL10 (2.6-fold, p<0.001). POLE EDMs also demonstrated striking upregulation of the T follicular helper gene CXCL13 (twofold, p=0.046) and regulatory T cell gene CTLA4 (1.3-fold, p=0.015). Upregulation of most of these genes in tumors has been shown to predict good prognosis.^27 28^

**Figure 2:**
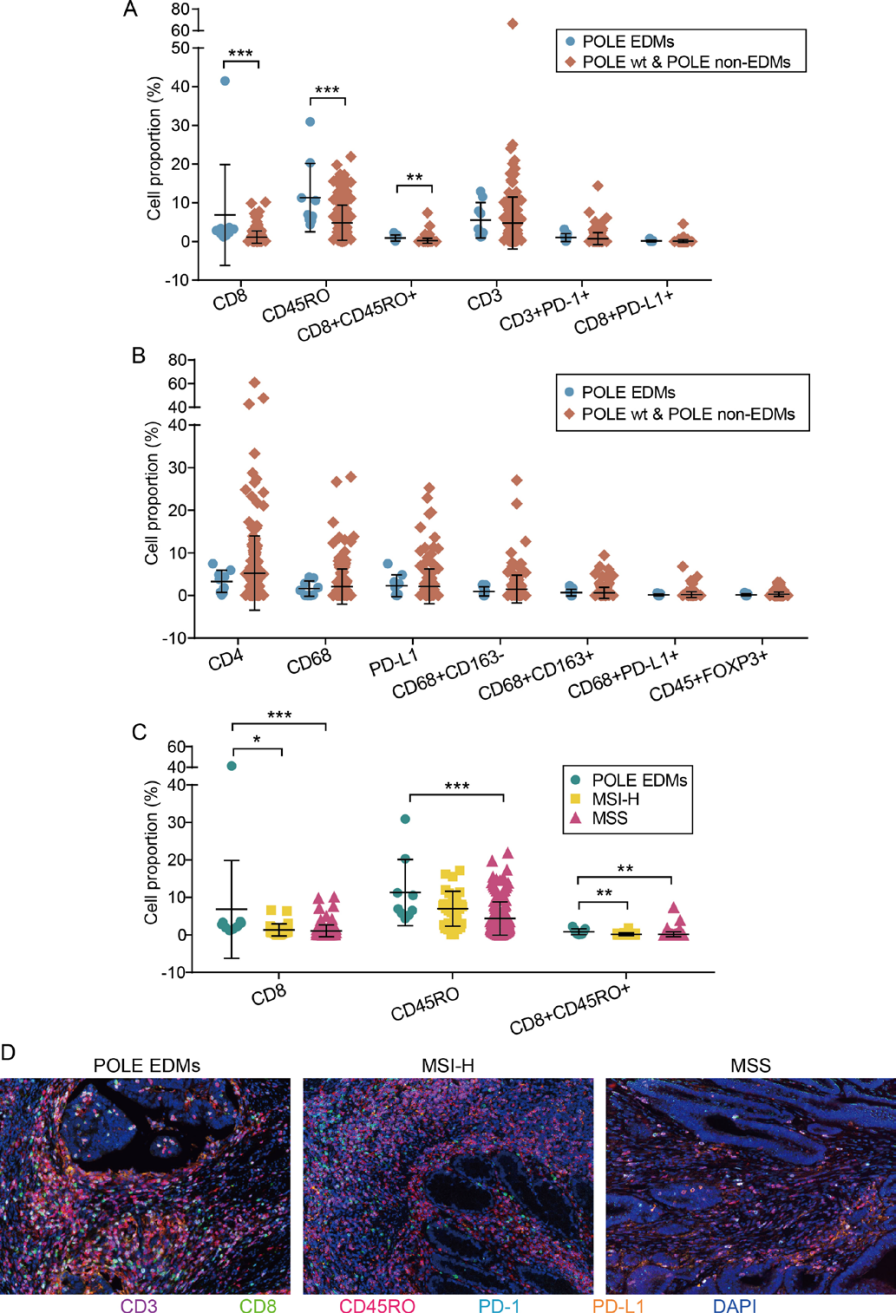
Five-color immunohistochemical multiplex analysis of stage II CRCs based on pole and MSI status. (A) Comparisons of cell proportion of immune cells (CD8+, CD45RO+, CD8 +CD45RO+, CD3+, CD3 +PD-1+ and CD8+PD-L1+) between pole EDMs and pole wild type or non-EDMs (panel 1). (B) Comparisons of cell proportion of immune cells (CD4+, CD68+, PD-L1+, CD68+CD163-, CD68+CD163+, CD68+PD-L1+ and CD+FOXP3+) between pole EDMs and pole wild-type or non-EDMs (panel 2). (C) Comparisons of cell proportion of immune cells (CD8+, CD45RO+ and CD8+CD45RO+) between pole EDMs, MSI-H and MSS. (D) Representative immunohistochemical multiplex images of CD8+, CD45RO+ and CD8+CD45RO+ in POLE EDMs, MSI-H and MSS tumors. *P<0.05, **P<0.01, ***P<0.001. CRCs, colorectal cancer; EDMs, exonuclease domain mutations; MSI-H, microsatellite instability-high; MSS, microsatellite stable.

In order to better understand tumor immune infiltration microenvironment of POLE EDMs in stage II CRCs, immune cells were quantified and tumor-infiltrating lymphocyte (TIL) ratios were subsequently calculated. As shown in figure 2A, B and a trend of increases of CD8+ cytotoxic T lymphocytes (CTL) (p<0.001), CD45RO+ memory immune cell (MIC) (p<0.001) and CD8 +CD45RO+MIC (p=0.003) in POLE EDMs was observed compared with POLE wild-type or non-EDMs. Then combined with MSI status, we found that the MSI-H tumors appear to have an intermediate phenotype, having lower expression of CD8+CTL, CD45RO+MIC, and CD8+CD45RO+MIC+ than POLE EDMs tumors, but higher than MSS tumors (figure 2C and D). Collectively, these findings may prove that compared with other stage II CRCs, POLE EDMs had greater T lymphocyte infiltration capable of exerting antitumor activity.

### Survival analysis for MSI status and POLE mutations

**Figure 3:**
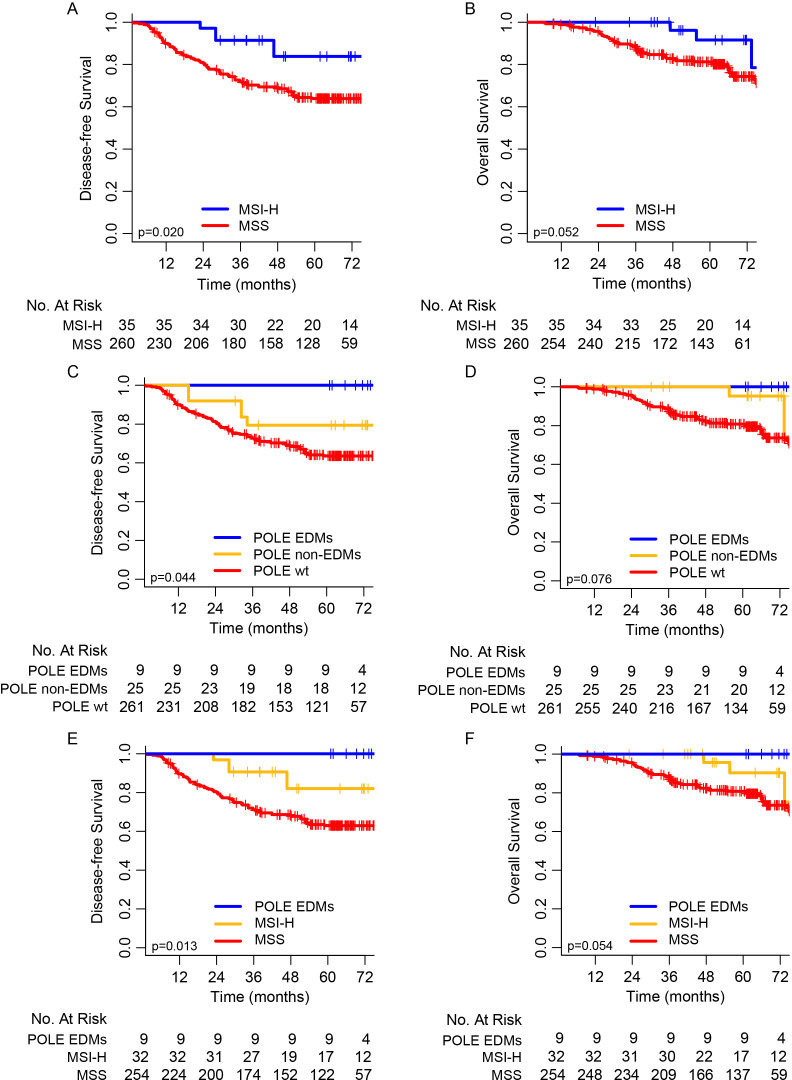
Kaplan-Meier curves of disease-free survival (DFS) and overall survival (OS). (A, B) Kaplan-Meier curves of DFS and OS between MSI-H and MSS stage II CRCs patients. (C, D) Kaplan-Meier curves of DFS and OS among stage II colorectal cancer patients with pole EDMs, pole non-EDMs and pole wild type. (E, F) Kaplan-Meier curves of DFS and OS among stage II colorectal cancer patients with pole EDMs, MSI-H, and MSS. CRCs, colorectal cancer; EDMs, exonuclease domain mutations; MSI-H, microsatellite instability-high; MSS, microsatellite stable.

We examined the association of MSI status and POLE mutations with clinical outcomes (DFS and OS). Considering all 295 patients, the 5 years DFS and OS rates for patients with MSI-H were 83.8% and 91.6%, respectively, compared with 63.9% and 81.2% for patients with MSS (p=0.020 for 5 year DFS and p=0.052 for 5 year OS, figure 3A and B).

Based on the sequencing results of the entire gene coding sequences, POLE mutations were classified into three subgroups: wild-type, non-EDMs and EDMs. In our series, none of the nine patients with POLE EDMs had recurrence, while 5 of 25 patients (20.0%) with POLE non-EDMs and 90 of 261 patients (34.4%) with POLE wild-type had local or distant recurrences. The 5 years DFS rates were 100%, 79.5% and 63.6% for patients with POLE EDMs, non-EDMs, and wild-type, respectively. Patients with POLE EDMs had significantly higher 5 years DFS rate than that of patients with POLE non-EDMs or POLE wild-type (p=0.044), and similar 5 years DFS rates were found between patients with POLE non-EDMs and POLE wild-type (figure 3C). Although the 5 years OS rates were not significantly different among the three groups, a similar trend was also observed (figure 3D). By comparing the POLE EDMs with mutations of other 35 genes in the ColonCore panel, POLE EDMs were related to mutations of PTEN, PIK3CA, TP53 and MMR genes, but the survival differences of POLE EDMs were not related to mutations of other genes tested in our study (online supplementary table 5).

10.1136/jitc-2020-000881.supp12 Supplementary data

Furthermore, considering the excellent outcomes of POLE EDMs, we classified all patients with stage II CRCs into three groups: POLE EDMs (regardless of MSI status), MSI-H and MSS. Significant difference of outcomes was observed among the three groups. The 5 years DFS rates were 100% in the group of POLE EDMs, 82.0% in the group of MSI-H and 63.0% in the group of MSS, with a significant p value of 0.013 among the three groups (figure 3E); the 5 years OS rates were 100% in the group of POLE EDMs, 90.3% in the group of MSI-H and 80.8% in the group of MSS, with a clear trend and marginal significant p value (figure 3F).

## Discussions

In our study, we used NGS technology to evaluate the detailed mutation profile and prognostic value of POLE gene in stage II CRC. Then, using a five-color immunohistochemical multiplex technique, we aimed to figure out immune cell infiltration in POLE EDMs tumors. Our findings have shown that POLE mutation at its exonuclease (proofreading) domains exhibited distinct molecular features and manifested excellent treatment outcomes for patients with stage II CRC. Using NGS technology, we could study the mutation features of all exons of POLE gene, therefore, extending the analysis beyond the exonuclease domain. We found somatic mutations of POLE in 11.5% of all patients altogether. However, the mutations at exonuclease domain were at low frequency (3.1% for POLE EDMs) in stage II cancers. POLE EDMs were previously reported only in 1%–2% of patients with CRC, this discrepancy may be due to different testing techniques and domains sequenced. By reviewing the POLE mutations in three different published CRC cohorts that used NGS technology, POLE mutations were detected in 7% (67/967) of patients, and POLE EDMs were only detected in 2.3% (22/967) of patients with CRCs.^29^ In a study with large number of patients, Domingo *et al* 25 reported 1.0% of POLE EDM in patients with stage II/III CRC. However, they only analyzed 3–4 target mutation sites by allele-specific PCR or Sanger sequencing, which may underestimated the real frequency of POLE EDMs. Even though, 62.1% of POLE EDMs in their study was detected in stage II CRCs, compared with 24.2% in stage III CRCs (p=0.003). Consistent with previous studies, the reported common mutation sites (P286R, V411L and S459F) were only detected in four patients (1.5%) in our series. Besides, we also found other six mutations in POLE exonuclease domains, therefore, NGS was necessary to thoroughly assess the mutations of POLE at all exonuclease domains.

In this study, we found that 33% of POLE EDMs positive patients were classified as MSI-H when assessed by NGS.^30^ Intriguingly, only one of these cases was classified as dMMR by IHC. One explanation for such difference is that, in the two cases with MSI-H and pMMR status, the elevated MSI may be caused by the deficiency of other genes or proteins of the MMR system. Most of studies reported that POLE EDMs were detected in pMMR patients,^25 31 32^ in our study, the only one patient with dMMR had no somatic mutations in any of MMR genes but the dMMR was caused by methylation of hMLH1, which may suggested a distinct molecular process of these patient.

Previous studies found the mutation in POLE exonuclease domain may cause disorder of DNA replication and tumor hypermutation.^33^ In our study, we further tested somatic mutations of 520 genes for the nine patients with POLE EDMs. All patients tested had high TMB. Thus, the results of our study were concordant to previous studies.^34 35^ Moreover, Domingo *et al* 25 also reported the upregulation of immune checkpoint in patients with POLE EDMs. These findings further suggested that patients with POLE EDM may be good candidate for immune checkpoint inhibitors in CRCs when clinically indicated.

By complementary analysis of the correlation between POLE EDMs and expression of immune related genes in TCGA series, we found that POLE EDMs are positive correlation with a striking CD8+ lymphocytic infiltrate, a gene signature of T cell infiltration, and marked upregulation of cytotoxic T cell effector markers. These findings indicated that POLE EDMs cases are characterized by unique immune response microenvironment. Further five-color immunohistochemical multiplex technique was conducted to demonstrated that a trend of increases of CD8+ CTL, CD45RO+MIC, and CD8 +CD45RO+MIC in POLE EDMs were observed. Collectively, these findings strengthened the conclusion that POLE EDMs in stage II CRCs had greater T lymphocyte infiltration capable of exerting anti-tumor activity, which may explain the favorable outcome of these tumors.

For stage II CRC, it is still a great challenge for clinicians to choose the optimal adjuvant treatment modalities. A variety of studies were conducted to find helpful prognostic factors trying to improve clinically actions. Clinical factors, such as T4 disease, high-grade tumor, were widely used but also widely challenged clinically.^10 12^ With the development and application of molecular biology, more biomarkers were studied in stage II CRC in the last two decades. However, only MSI status (or IHC-assessed MMR status) was widely accepted as a helpful biomarker for stage II CRCs. Patients with MSI-H were confirmed harboring better outcomes and resistant to 5-FU based chemotherapy. In our study, we also found similar survival benefit for patients with MSI-H.

The prognostic effect of POLE EDMs has been rarely studied. Domingo *et al* 25 found that POLE EDM correlated to good prognosis for stage II/III CRC in a large number of series. However, the technique they used was only able to assess several target mutations within the exonuclease domain. The prognostic effect of all mutations in POLE exonuclease domain is still unknown to us. In addition, the authors also confirmed in the subgroup analysis that the prognostic effect of POLE EDMs was only significant in stage II CRCs. To our knowledge, our study was the first study focusing on the prognostic effect of mutations in all the exonuclease domain of POLE genes by NGS in stage II CRC. Although at a low frequency, excellent prognosis was found in patients with POLE EDMs. All patients with POLE EDMs maintained survival with free of disease by long-term follow-up, even with poor clinical factors in some patients. Similarly, there was also only one recurrence observed in POLE EDMs group in Domingo’s study.^25^ By combining the POLE mutation and MSI status, we further classified patients with stage II CRCs into three groups with significantly different outcomes. Patients with POLE EDMs, regardless of MSI status (or IHC-assessed MMR status), had best outcomes, suggesting the possibility of obviating adjuvant treatment even at clinical high risk for patient belonging to this group.

Moreover, we also confirmed that patients with POLE mutations at non-exonuclease domain had no better outcome compared with patient with POLE wild type. These findings showed the importance of discriminating different POLE mutations in the sequencing report to help clinical decision making. It is also helpful for design accurate and cost-effective NGS targeted panels.

Our study had several limitations. First, the total number of patients with POLE EDMs was small and additional studies are required to establish if the results could be widely used is still unknown to us, especially when patients had concurrent clinically high-risk factors, such as T4 disease. Second, six out of nine patients with POLE EDM received adjuvant chemotherapy, therefore, it should be concluded with caution that stage II patients with POLE EDM could obviate any adjuvant treatment. Third, as all patients with POLE EDMs were disease free at last follow-up, multivariate analysis was not able to be conducted in our series. Although the clinicopathological factors were relatively well balanced between relapse group and no-relapse group, further studies were still needed to confirm our observations.

In conclusion, characterized by distinct clinicopathological and molecular features, ultramutated POLE EDMs could be detected in a small subset of stage II CRCs with extremely high TMB. Patients with POLE EDMs had excellent outcomes in stage II CRCs, regardless of MSI status. Sequencing of all the exonuclease domain of POLE gene is recommended in clinical practice.

## Methods

The detailed methods could be found in online supplemental file 13. (See also online supplementary figure 6 and and online supplementary tables 6–8).

10.1136/jitc-2020-000881.supp15 Supplementary data

10.1136/jitc-2020-000881.supp6 Supplementary data

10.1136/jitc-2020-000881.supp13 Supplementary data

10.1136/jitc-2020-000881.supp14 Supplementary data

10.1136/jitc-2020-000881.supp7 Supplementary data
