# Supplementary figures and images for: Somatic POLE exonuclease domain mutations elicit enhanced intratumoral immune responses in stage II colorectal cancer
Source: J Immunother Cancer. 2020 Aug 27;8(2):e000881. doi: 10.1136/jitc-2020-000881 (PMC7454238; doi:10.1136/jitc-2020-000881)

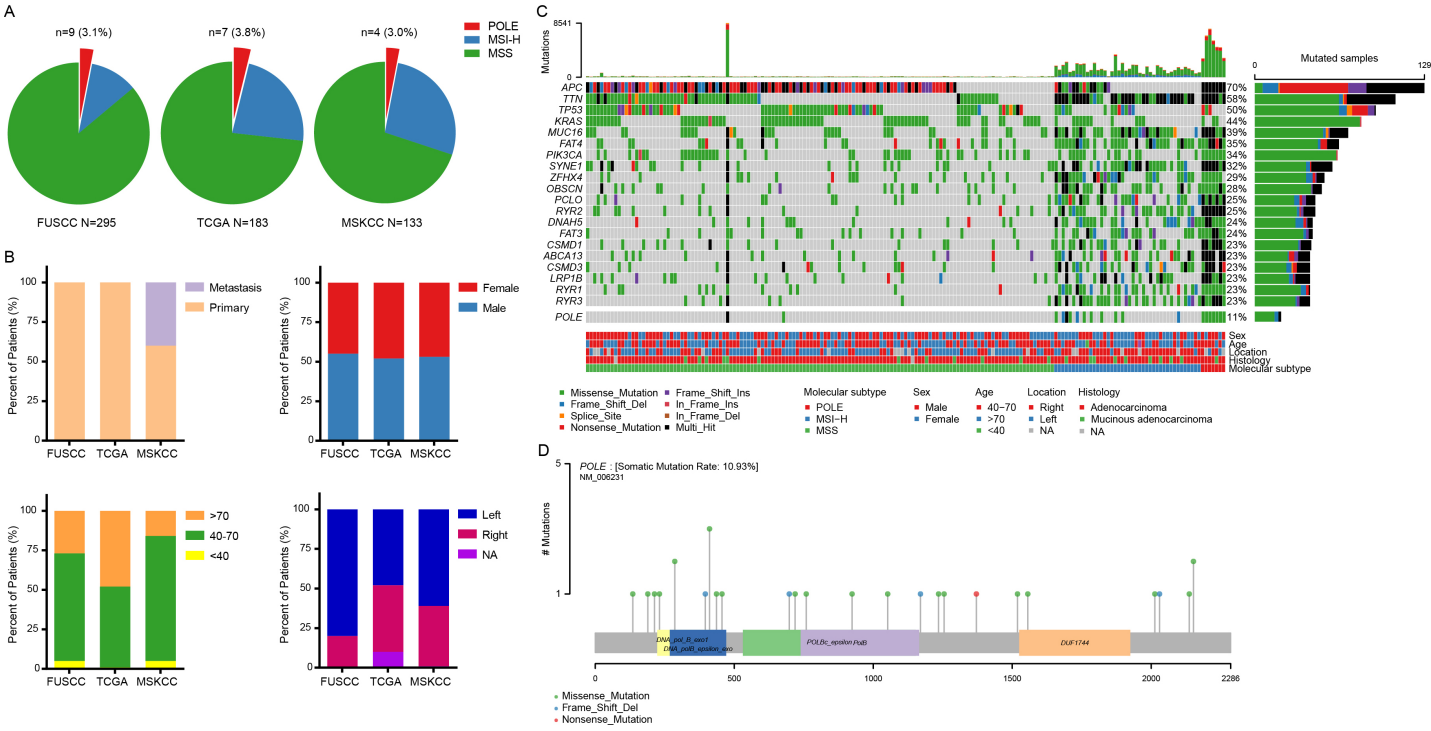

Supplement: Supplementary data [file jitc-2020-000881supp001.pdf]

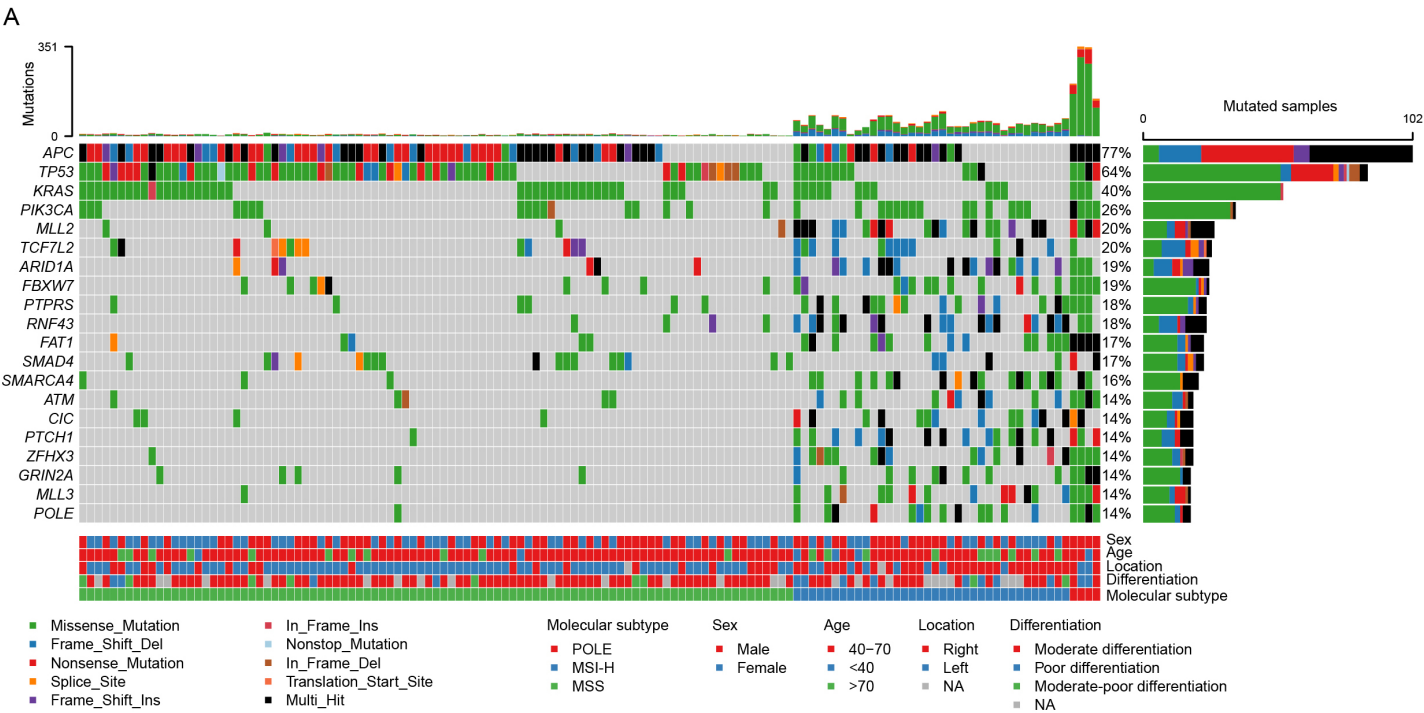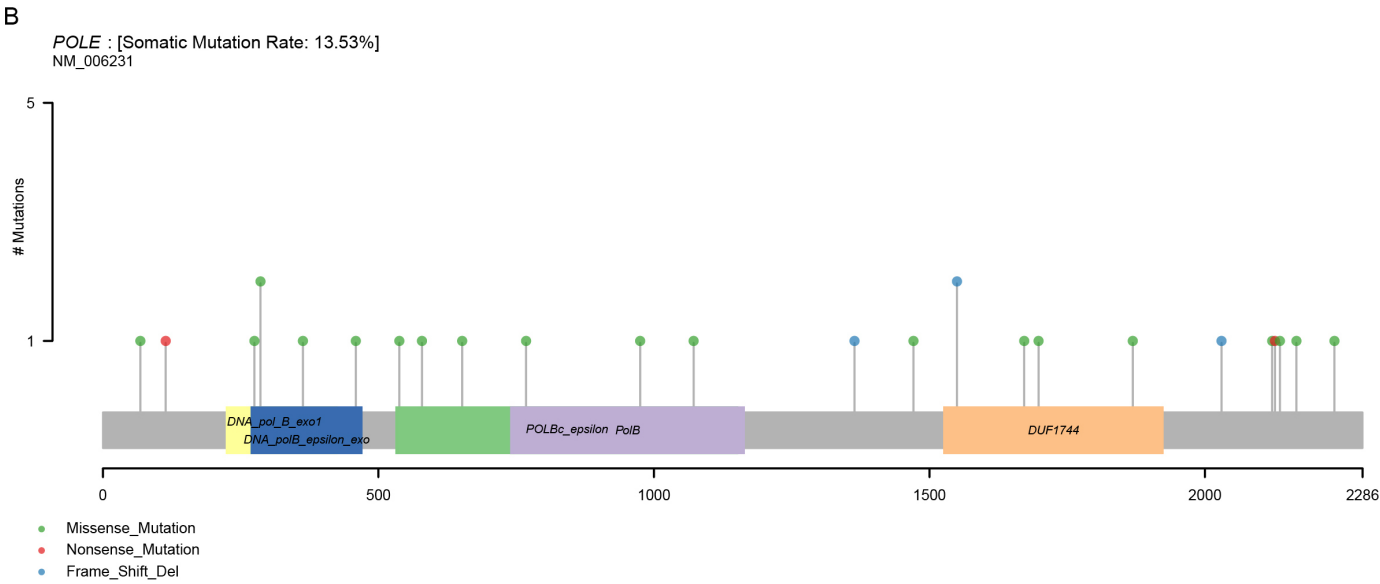

Supplement: Supplementary data [file jitc-2020-000881supp002.pdf]

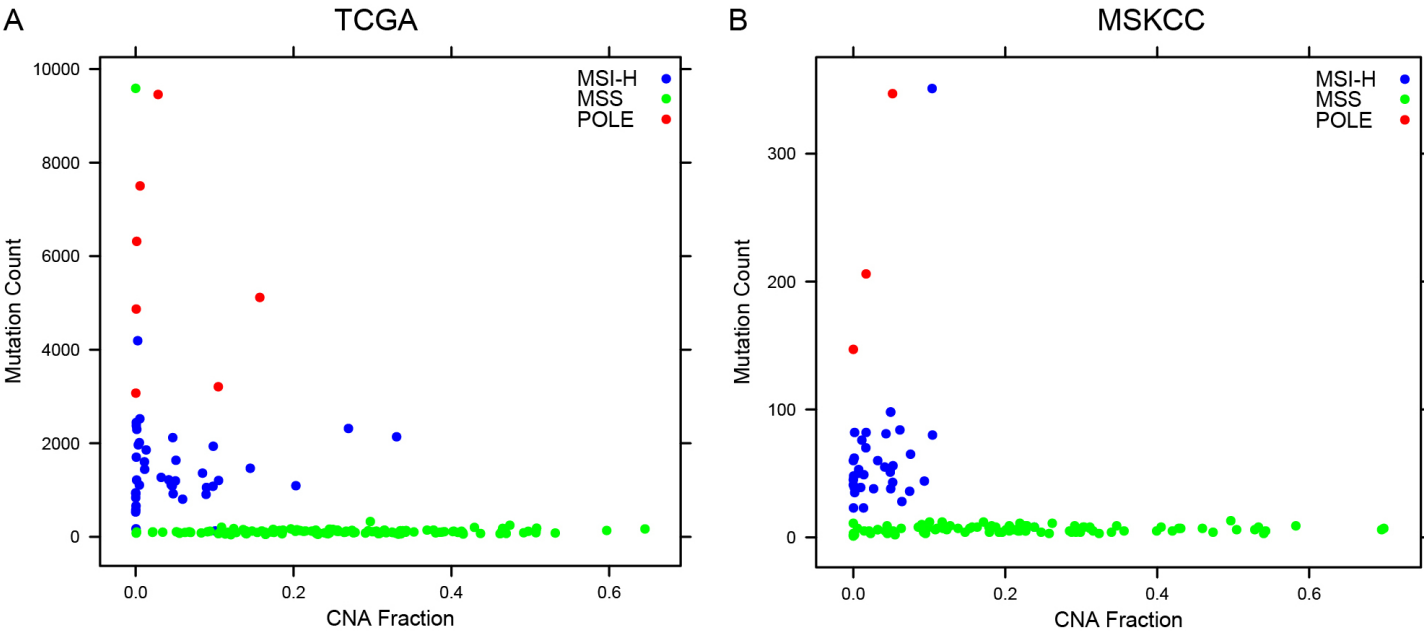

Supplement: Supplementary data [file jitc-2020-000881supp005.pdf]

A

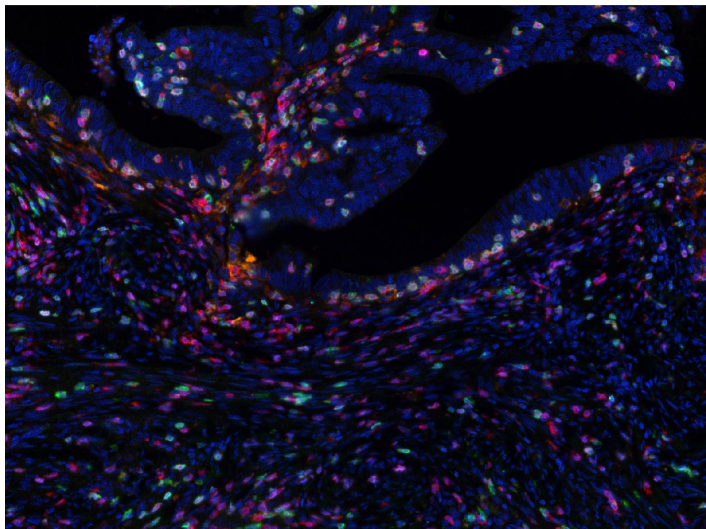

CD3 CD8 CD45RO PD-1 PD-L1 DAPI

B

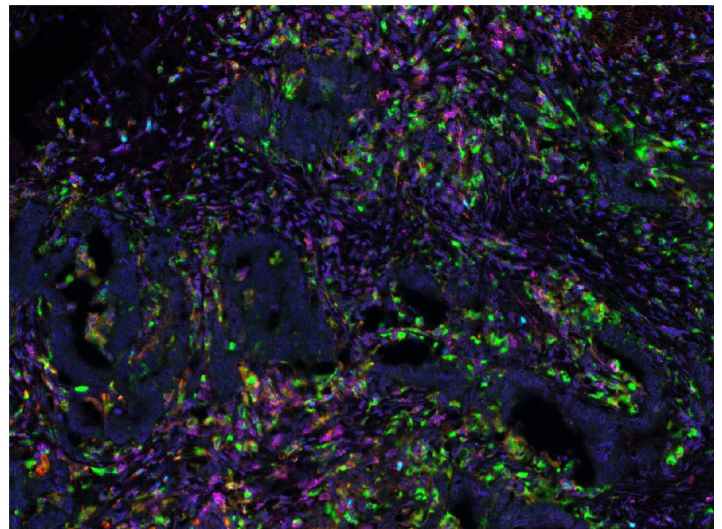

CD4 CD68 CD163 FOXP3 PD-L1 DAPI

Supplement: Supplementary data [file jitc-2020-000881supp006.pdf]
