# Supplementary material for: Somatic POLE exonuclease domain mutations elicit enhanced intratumoral immune responses in stage II colorectal cancer
Source: J Immunother Cancer. 2020 Aug 27;8(2):e000881. doi: 10.1136/jitc-2020-000881 (PMC7454238; doi:10.1136/jitc-2020-000881)
Supplement: Supplementary data [file jitc-2020-000881supp008.pdf]

Supplementary Table 2. Demographic and clinicopathological characteristics according to stage II CRC POLE status in MSKCC cohort.

Characteristic	POLE EDMs	POLE wild-type & POLE non-EDMs	p value
Total	4 (3.0)	129 (97.0)	--
Age (IQR)	47 (31-59)	59 (50-67)	0.071
Gender			0.054
Female	0 (0)	63 (48.8)	
Male	4 (100)	66 (51.2)	
Tumor grade			0.063
Moderate differentiation	2 (50.0)	83 (64.3)	
Moderate-poor differentiation	0 (0)	9 (7.0)	
Poor differentiation	2 (50.0)	12 (9.3)	
Unknown	0 (0)	25 (19.4)	
Location			0.856
Right	2 (50.0)	48 (37.2)	
Left	2 (50.0)	79 (61.2)	
Unknown	0 (0)	2 (1.6)	
Sample type			0.098
Primary	4 (100)	76 (58.9)	
Metastasis	0 (0)	53 (41.1)	
MSI status			0.898
MSS	3 (75.0)	93 (72.1)	
MSI-H	1 (25.0)	36 (27.9)	

CRC, colorectal cancer; EDM, exonuclease domain mutation; IQR, interquartile range; MSI, microsatellite instability; MSS, microsatellite stabilization
