# Supplementary material for: Somatic POLE exonuclease domain mutations elicit enhanced intratumoral immune responses in stage II colorectal cancer
Source: J Immunother Cancer. 2020 Aug 27;8(2):e000881. doi: 10.1136/jitc-2020-000881 (PMC7454238; doi:10.1136/jitc-2020-000881)
Supplement: Supplementary data [file jitc-2020-000881supp009.pdf]

Supplementary Table 3. Somatic POLE EDMs by NGS in stage II CRC.

NGS-MSI status	POLE EDMs				
	Sample ID	Exon rank	Mutation	TMB	MMR-IHC
POLE EDMs					
NGS-MSS	RS1724514FFP	9	POLE_p.P286R;	499.2	pMMR
NGS-MSS	RS1724574FFP	9	POLE_p.P286R;	204.8	pMMR
NGS-MSS	RS1724507FFP	9	POLE_p.S297F	446.8	pMMR
NGS-MSS	RS1711472FFP	13	POLE_p.V411L	174.6	pMMR
NGS-MSS	RS1724566FFP	14	POLE_p.S459F	96	pMMR
NGS-MSS	RS1724590FFP	14	POLE_p.A463P	54	pMMR
NGS-MSI-H	RS1725783FFP	10	POLE_p.E311G	105.6	pMMR
NGS-MSI-H	RS1724602FFP	11	POLE_p.G364W	81	dMMR
NGS-MSI-H	RS1711447FFP	13	POLE_p.R413M; POLE_p.A448T	145.2	pMMR

EDM, exonuclease domain mutation; NGS, next-generation sequencing; CRC, colorectal cancer; MSI, microsatellite instability; MSS, microsatellite stabilization; TMB, tumor mutation burden; IHC, immunohistochemical; pMMR, proficient mismatch repair; dMMR, deficient mismatch repair
