# Supplementary material for: Somatic POLE exonuclease domain mutations elicit enhanced intratumoral immune responses in stage II colorectal cancer
Source: J Immunother Cancer. 2020 Aug 27;8(2):e000881. doi: 10.1136/jitc-2020-000881 (PMC7454238; doi:10.1136/jitc-2020-000881)
Supplement: Supplementary data [file jitc-2020-000881supp010.pdf]

Supplementary Table 4. List of 22 mutations analyzed in POLE gene.

Patient ID	Exon number	Mutations
**FUSCC**		
RS1724514FFP	9	p.P286R
RS1724574FFP	9	p.P286R
RS1724507FFP	9	p.S297F
RS1711472FFP	13	p.V411L
RS1724566FFP	14	p.S459F
RS1724590FFP	14	p.A463P
RS1725783FFP	10	p.E311G
RS1724602FFP	11	p.G364W
RS1711447FFP	13, 14	p.R413M; p.A448T
**TCGA**		
TCGA-AA-A010	13	p.P436R
TCGA-AA-A00N	13	p.V411L
TCGA-AA-3984	13	p.V411L
TCGA-CA-6718	9	p.P286R
TCGA-AZ-4315	13	p.V411L
TCGA-F5-6814	9	p.P286R
TCGA-AA-3510	14	p.A456P
**MSKCC**		
P-0004865	9	p.P286R
P-0005824	9	p.P286R
P-0006960	11	p.N363D
P-0011357	9, 14	p.D275G; p.S459F
