# Supplementary material for: Somatic POLE exonuclease domain mutations elicit enhanced intratumoral immune responses in stage II colorectal cancer
Source: J Immunother Cancer. 2020 Aug 27;8(2):e000881. doi: 10.1136/jitc-2020-000881 (PMC7454238; doi:10.1136/jitc-2020-000881)
Supplement: Supplementary data [file jitc-2020-000881supp011.pdf]

Supplementary Table 5. Survival differences related to mutations of genes associated with CRC

Variable	5-year DFS	
	Log rank χ^2	P-value
RAS	0.159	0.690
BRAF	2.071	0.150
TP53	1.454	0.228
APC	0.356	0.551
PIK3CA	1.611	0.204
PTEN	0.001	0.980
MUTYH	0.559	0.455
EpCAM	0.117	0.732
ERBB2	0.263	0.608
SMAD4	0.053	0.818
MLH1	0.141	0.707
MSH2	0.047	0.829
MSH6	0.210	0.647
PMS2	0.294	0.588

CRC, colorectal cancer; DFS, disease-free survival
