# Supplementary material for: Somatic POLE exonuclease domain mutations elicit enhanced intratumoral immune responses in stage II colorectal cancer
Source: J Immunother Cancer. 2020 Aug 27;8(2):e000881. doi: 10.1136/jitc-2020-000881 (PMC7454238; doi:10.1136/jitc-2020-000881)

Figure 3S The MSI assessment by NGS for 3 patients with POLE mutations at exonuclease domains (the red bars are tumors, and the green bars are normal control)

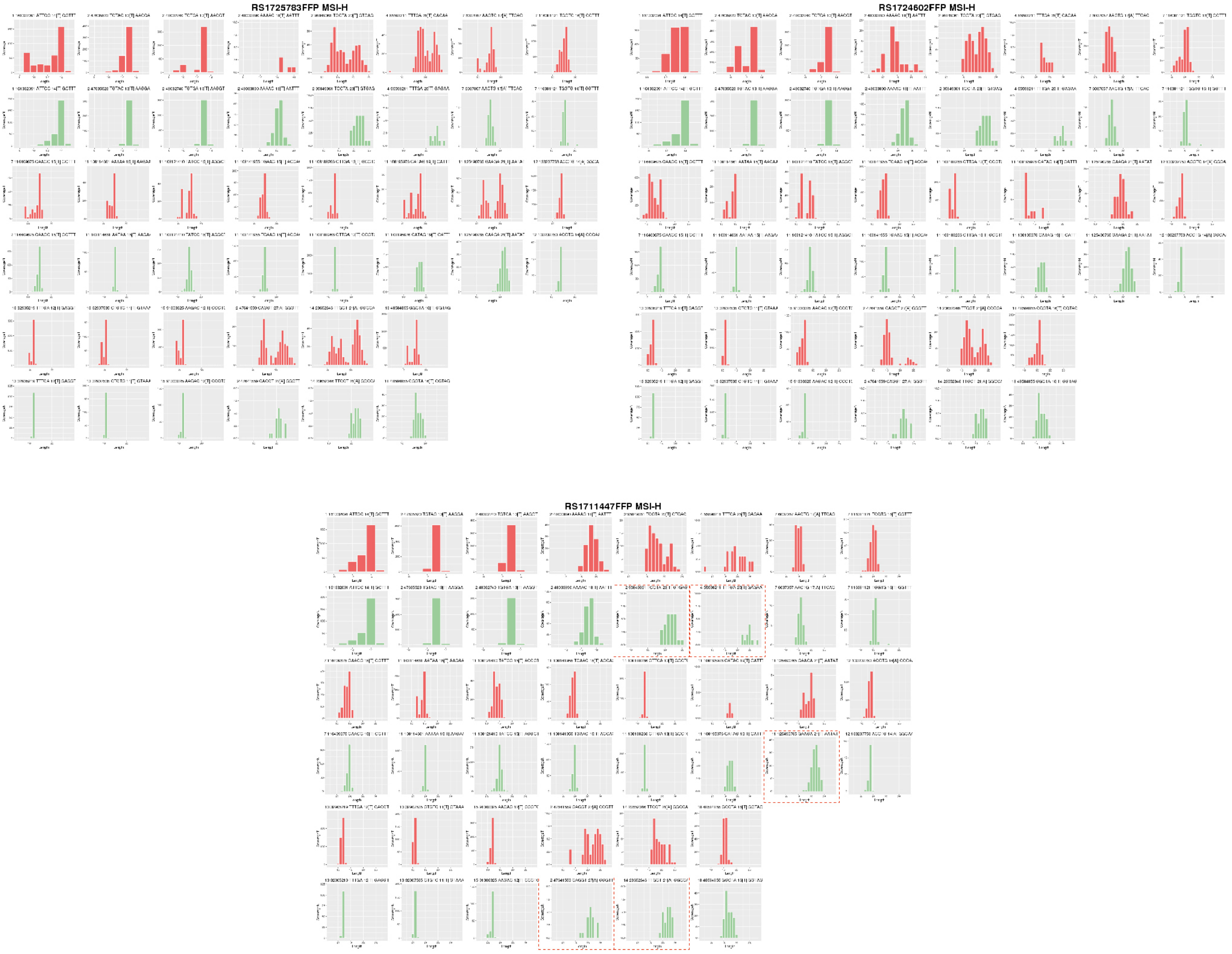

Supplement: Supplementary data [file jitc-2020-000881supp003.pdf]
