# Supplementary material for: Somatic POLE exonuclease domain mutations elicit enhanced intratumoral immune responses in stage II colorectal cancer
Source: J Immunother Cancer. 2020 Aug 27;8(2):e000881. doi: 10.1136/jitc-2020-000881 (PMC7454238; doi:10.1136/jitc-2020-000881)
Supplement: Supplementary data [file jitc-2020-000881supp004.pdf]

Figure 4S. Methylation of hMLH1 gene in sample with POLE mutation and IHC-assessed dMMR.

Sequencing block (containing 5 CpG sites in total):

agagCggaCagCgatCtCtaaCgCgCaagCgCa

Normal sample:

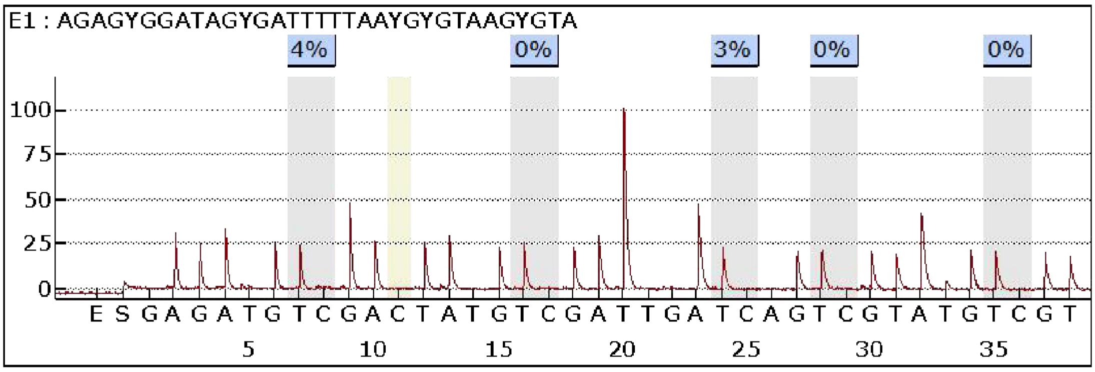

Sample with POLE mutation and IHC-assessed dMMR:

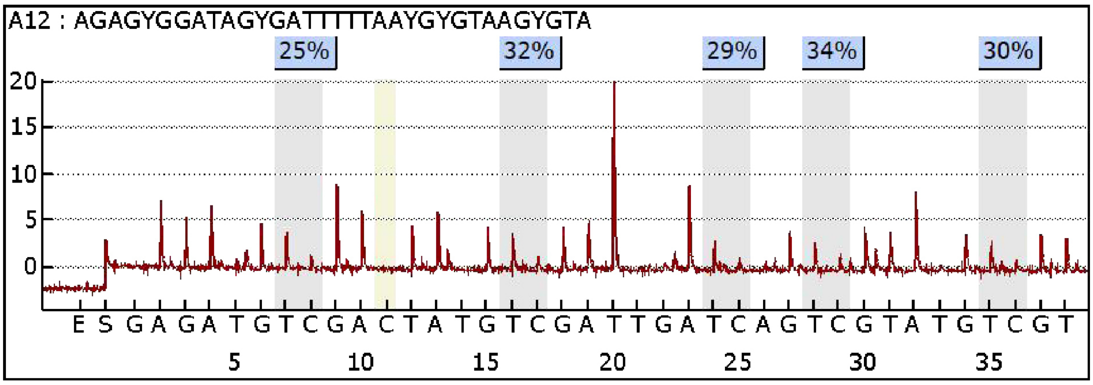

Average methylation level of hMLH1 gene: **30%** (Reference methylation level of hMLH1 gene≤6%)
