# Supplementary material for: Somatic POLE exonuclease domain mutations elicit enhanced intratumoral immune responses in stage II colorectal cancer
Source: J Immunother Cancer. 2020 Aug 27;8(2):e000881. doi: 10.1136/jitc-2020-000881 (PMC7454238; doi:10.1136/jitc-2020-000881)
Supplement: Supplementary data [file jitc-2020-000881supp012.pdf]

Supplementary Table 6. 37 CRC-related genes in ColonCore panel.

TP53
KRAS
MSH6
MUTYH
EPCAM
MET
ERBB2
PIK3CA
BRAF
PTEN
APC
POLE
POLD1
BRCA1
GALNT12
ATM
KIT
CDH1
PMS1
BRCA2
BMPR1A
STK11
MLH1
PTCH1
EGFR
SMAD4
NRAS
PDGFRA
SDHD
BLM
GREM1
AKT1
HRAS
MSH2
CHEK2
PMS2
SDHB
