# Supplementary material for: Somatic POLE exonuclease domain mutations elicit enhanced intratumoral immune responses in stage II colorectal cancer
Source: J Immunother Cancer. 2020 Aug 27;8(2):e000881. doi: 10.1136/jitc-2020-000881 (PMC7454238; doi:10.1136/jitc-2020-000881)
Supplement: Supplementary data [file jitc-2020-000881supp015.pdf]

Materials and methods

Patients selection and clinicopathological characteristics

Patients with stage II CRC were retrospectively collected from the institutional database of Fudan University Shanghai Cancer Center (FUSCC), who were treated with curative intent between January 2008 and December 2013. All patients underwent radical resection of primary tumor and were pathologically confirmed adenocarcinoma with stage II CRC. Exclusion criteria included: 1) non-stage II CRC, 2) undergoing neoadjuvant chemotherapy or chemoradiotherapy, 3) loss of follow-up at the first 12 months after surgery, 4) detection of distant metastasis within 6 months after surgery, 5) loss of assessable clinicopathological or treatment information, and 6) insufficient formalin-fixed, paraffin-embedded (FFPE) tissue for study, 7) refusal of informed consent.

Records on the following clinicopathological and molecular variables were extracted from FUSCC dataset: gender; age at diagnosis; pretreatment carcinoembryonic antigen (CEA); primary tumor site; histological type; grade of differentiation; number of lymph nodes harvested; depth of intestinal wall invasion; lymphovascular invasion; perineural invasion; and adjuvant chemotherapy. T stage and N stage were determined by the American Joint Committee on Cancer (AJCC) Cancer Staging Manual (7th edition)[1].

Postoperative follow-up was conducted according to hospital routines. Briefly, CEA and CA19-9 were tested every 3-6 months at the first 3 years and every 6 months thereafter till 5 years. Chest X ray or CT scans and abdominoperineal CT scans with

contrast or MRI were performed every 6 months at the first 3 years and each year thereafter till 5 years. Additional blood or imaging test were performed when clinically indicated. The treatment and outcome information were collected by phone and from out-patient records.

The Ethical Committee and Institutional Review Board of our cancer center reviewed and approved this study protocol. All patients in the study willingly signed written informed consent before their surgical treatment.

TCGA and MSKCC data

The TCGA and MSKCC data of CRC tissues with gene mutations or expression levels, including clinicopathological information, were downloaded from TCGA (https://tcga-data.nci.nih.gov/) database and the cBioPortal for Cancer Genomics (https://www.cbioportal.org/).

Tissue management and sequencing

Sequencing was performed with hybridization capture-based NGS assay. Genomic DNA from formalin-fixed paraffin-embedded (FFPE) primary or metastatic colorectal tumors and patient-matched normal tissues were extracted using the Qiagen QiaAmp kit (Qiagen,Valencia, CA). Extracted DNA was sheared using the Covaris M220 instrument (Covaris, Woburn, MA). Custom DNA probes were designed for targeted genetic panel. DNA was hybridized with the capture probes baits. Sequencing libraries were prepared using commercial kit. Pooled libraries containing captured DNA fragments were sequenced using NextSeq 500 sequencer with pair-end reads.

Sequencing data analysis

Sequence data was mapped to the human genome (hg19) using BWA aligner v0.7.10. Local alignment optimization, variant calling and annotation were performed using GATK 3.2, MuTect, and VarScan. Variants were filtered using the VarScan fpfilter pipeline, with loci with depth less than 100 filtered out. At least 5 and 8 supporting reads were needed for INDELs and SNVs to be called in tissue samples, respectively. According to the ExAC, 1000 Genomes, dbSNP, ESP6500SI-V2 database, variants with population frequency over 0.1% were grouped as SNP and excluded from further analysis. Remaining variants were annotated with ANNOVAR and SnpEff v3.6. DNA translocation analysis was performed using both Tophat2 and Factera 1.4.3. For MSI status, reads aligned to the loci at every possible repeat length were counted using the same strategy as proposed by MSIsensor.

Test of NGS-based MSI and IHC-based MMR

ColonCore panel (Burning Rock, Guangzhou, China) is designed for simultaneous detection of MSI status and mutations in 37 CRC-related genes, including KRAS, NRAS, BRAF, hereditary CRC genes, and other genes related to carcinogenesis and tumor development (Supplementary Table 6). The MSI phenotype detection method of MSI-ColonCore was reported in previous study[2]. Briefly, it was a read-count-distribution-based approach. It utilized the coverage ratio of a specific set of repeat lengths as the main characteristic of each microsatellite locus, and categorized a locus as unstable if the coverage ratio was less than a given threshold. The MSI status of a sample was determined by the percentage of unstable loci in the given sample.

Immunohistochemistry staining was performed to examine the expression of four

MMR proteins, MLH1, MSH2, MSH6, and PMS2 on FFPE tissue. Primary monoclonal antibodies against MLH1 (clone ES05, diluted 1:50 [DAKO, Carpinteria, CA]), MSH2 (clone FE11, diluted 1:50 [Oncogene Research Products, Boston, MA]), MSH6 (clone EP49, diluted 1: 150 [DAKO, Carpinteria, CA]), and PMS2 (clone EP51, diluted 1: 50 [DAKO, Carpinteria, CA]) were used with external controls. Deficient MMR (dMMR) was interpreted when any of these MMR proteins was absent in the nuclear staining of tumor tissue while present in nuclear staining of adjacent normal tissue. The presence of expressions of all four proteins was considered proficient MMR (pMMR). IHC staining was assessed by two independent pathologists, and only concordant samples were included in the present study.

Tumor mutation burden estimation

All patients with somatic mutations of POLE exonuclease domains were further analyzed by a larger NGS-based gene panel (OncoScreen ^{plus}, Burning Rock, Guangzhou, China), which consisted 520 cancer related genes (Supplementary Table 7), spanning 1.64MB of human genome. This analysis was used to estimate the TMB of POLE EDMs. TMB was defined as the number of somatic, coding, base substation and indels per megabase of genome examined. Fusions and copy number variations are not counted. Non-coding mutations are not counted. Synonymous mutations are counted in order to reduce sampling noise. All germline variants were filtered by paired adjacent normal sample or white blood cells.

Five-color immunohistochemical multiplex

Tumor immune microenvironment was examined by multiplex immunohistochemistry

(Genecast Precision Medicine Technology Institute, Beijing, China). FFPE tissues were sectioned into 4-μm-thick sections. The slides were deparaffinized in xylene, rehydrated, and washed in tap water before boiling in Tris-EDTA buffer (pH 9; 643901; Klinipath, Duiven, the Netherlands) for epitope retrieval/microwave treatment (MWT). Endogenous peroxidase was blocked using Antibody Diluent/Block (72424205; PerkinElmer, Massachusetts, USA). Protein blocking was performed using Antibody Diluent/Block. One antigen requires one round of labeling, including primary antibody incubation, secondary antibody incubation, and TSA visualization, followed by labeling of the next antibody. Information on of the primary antibodies and the multiplex staining reagents is provided in Supplementary Table 8. Different primary antibodies CD3+, CD8+, CD45RO+, PD-1+, PD-L1+ (Panel 1), and CD4+, FOXP3+, CD68+, CD163+, PD-L1+ (Panel 2) were applied through sequential rounds of staining. Next, incubation with Opal Ploymer HRP Ms+Rb (2414515; PerkinElmer, Massachusetts, USA) was performed at 37°C for 10 min. TSA visualization was performed with the Opal seven-color IHC Kit (NEL797B001KT; PerkinElmer, Massachusetts, USA), containing fluorophores DAPI, and TSA Coumarin system (NEL703001KT; PerkinElmer, Massachusetts, USA). MWT was performed to remove the Ab TSA complex with Tris-EDTA buffer (pH 9). TSA single stain slides were finished with MWT and counterstained with DAPI for 5 min and were enclosed in Antifade Mounting Medium (I0052; NobleRyder, Beijing, China).

Multispectral Imaging

Slides were scanned using the PerkinElmer Vectra (Vectra 3.0.5; PerkinElmer, Massachusetts, USA). Multispectral images were unmixed using spectral libraries built from images of single stained tissues for each reagent using the inform Advanced Image Analysis software (inForm 2.3.0; PerkinElmer, Massachusetts, USA). A selection of 5-10 representative original multispectral images was used to train the inForm software (tissue segmentation, cell segmentation, phenotyping tool, and positivity score). All the settings applied to the training images were saved within an algorithm to allow batch analysis of multiple original multispectral images of the same tissue[3]. The representative graphs of two panels were shown in Supplementary Figure 6.

Statistical analysis

We compared the patient demographics, pathologic characteristics, and gene mutations between POLE EDMs and wild-type stage II CRC patients using chi-squared tests and Fisher's exact tests. The percentages of tumor infiltrated immune cells in each tissue segmentation were obtained. And the data was used for next analysis. Differences of immune cell infiltration between groups were analyzed using a t test (normal distribution) or the Mann-Whitney U test (abnormal distribution) as appropriate. Survival curves were plotted using the Kaplan-Meier method with log-rank test. All statistical analyses were performed using R (version 2.15.0, www.r-project.org). All statistical tests were 2-sided, and $p<0.05$ was considered statistically significant.

BMJ Publishing Group Limited (BMJ) disclaims all liability and responsibility arising from any reliance placed on this supplemental material which has been supplied by the author(s)

Reference

1. Edge S, Byrd D, Compton C*, et al.* AJCC cancer staging manual (7th ed). New York, NY: Springer. 2010.
2. Zhu L, Huang Y, Fang X*, et al.* A Novel and Reliable Method to Detect Microsatellite Instability in Colorectal Cancer by Next-Generation Sequencing. ***J Mol Diagn*** 2018;20:225-231.
3. Gorris MAJ, Halilovic A, and Rabold K. Eight-Color Multiplex Immunohistochemistry for Simultaneous Detection of Multiple Immune Checkpoint Molecules within the Tumor Microenvironment. 2018;200:347-354.
