# Supplementary material for: Somatic POLE exonuclease domain mutations elicit enhanced intratumoral immune responses in stage II colorectal cancer
Source: J Immunother Cancer. 2020 Aug 27;8(2):e000881. doi: 10.1136/jitc-2020-000881 (PMC7454238; doi:10.1136/jitc-2020-000881)
Supplement: Supplementary data [file jitc-2020-000881supp013.pdf]

Supplementary Table 7. Gene list of 520 genes.

Gene	NGS	CNV	TransID
ABL1	wholeExon	Y	NM_005157.5
ABL2	selectedExon	Y	NM_007314.3
ACVR1	selectedExon	Y	NM_001105.4
ACVR1B	selectedExon	Y	NM_020328.3
ADGRA2	selectedExon	Y	NM_032777.9
AKT1	wholeExon	Y	NM_001014432.1
AKT2	wholeExon	Y	NM_001626.5
AKT3	wholeExon	Y	NM_005465.4
ALK	wholeExon/fusion	Y	NM_004304.4
ALOX12B	selectedExon	Y	NM_001139.2
ANKRD11	selectedExon	Y	NM_001256182.1
APC	wholeExon	Y	NM_000038.5
AR	wholeExon	Y	NM_000044.3
ARAF	wholeExon	Y	NM_001256196.1
ARID1A	wholeExon	Y	NM_006015.4
ARID1B	wholeExon	Y	NM_020732.3
ARID2	wholeExon	Y	NM_152641.2
ARID5B	selectedExon	Y	NM_032199.2
ASXL1	wholeExon	Y	NM_015338.5
ASXL2	selectedExon	Y	NM_018263.4
ATF1	selectedExon	Y	NM_005171.4
ATM	wholeExon	Y	NM_000051.3
ATR	wholeExon	Y	NM_001184.3
ATRX	wholeExon	Y	NM_000489.4
AURKA	wholeExon	Y	NM_001323303.1
AURKB	wholeExon	Y	NM_001284526.1
AXIN1	wholeExon	Y	NM_003502.3
AXIN2	selectedExon	Y	NM_004655.3
AXL	wholeExon	Y	NM_021913.4
B2M	selectedExon	Y	NM_004048.2
BAP1	wholeExon	Y	NM_004656.3
BARD1	wholeExon	Y	NM_000465.3
BCL2	wholeExon	Y	NM_000633.2
BCL2L1	wholeExon	Y	NM_001317919.1
BCL6	wholeExon	Y	NM_001130845.1
BCOR	wholeExon	Y	NM_001123383.1
BCORL1	selectedExon	Y	NM_021946.4
BCR	fusion	Y	NM_004327.3

BIRC3	selectedExon	Y	NM_001165.4
BLM	wholeExon	Y	NM_000057.3
BMPR1A	selectedExon	Y	NM_004329.2
BRAF	wholeExon/fusion	Y	NM_004333.4
BRCA1	wholeExon	Y	NM_007294.3
BRCA2	wholeExon	Y	NM_000059.3
BRD4	wholeExon	Y	NM_058243.2
BRIP1	wholeExon	Y	NM_032043.2
BTK	wholeExon	Y	NM_000061.2
CARD11	wholeExon	Y	NM_032415.5
CASP8	selectedExon	Y	NM_001228.4
CBFB	wholeExon	Y	NM_022845.2
CBL	wholeExon	Y	NM_005188.3
CCND1	wholeExon/SNPCNV	Y	NM_053056.2
CCND2	wholeExon	Y	NM_001759.3
CCND3	wholeExon	Y	NM_001760.4
CCNE1	wholeExon	Y	NM_001238.3
CD274	wholeExon/UTR	Y	NM_014143.3
CD79A	wholeExon	Y	NM_001783.3
CD79B	wholeExon	Y	NM_001039933.2
CDC73	wholeExon	Y	NM_024529.4
CDH1	wholeExon	Y	NM_004360.4
CDK12	wholeExon	Y	NM_016507.3
CDK4	wholeExon/SNPCNV	Y	NM_000075.3
CDK6	wholeExon/SNPCNV	Y	NM_001145306.1
CDK8	wholeExon	Y	NM_001260.2
CDKN1A	wholeExon	Y	NM_001291549.1
CDKN1B	wholeExon	Y	NM_004064.4
CDKN1C	wholeExon	Y	NM_000076.2
CDKN2A	wholeExon	Y	NM_000077.4
CDKN2B	wholeExon	Y	NM_004936.3
CDKN2C	wholeExon	Y	NM_001262.2
CEBPA	wholeExon	Y	NM_004364.4
CHD1	wholeExon	Y	NM_001270.2
CHD2	selectedExon	Y	NM_001271.3
CHD4	selectedExon	Y	NM_001273.3
CHEK1	wholeExon	Y	NM_001114121.2
CHEK2	wholeExon	Y	NM_007194.3
CHUK	selectedExon	Y	NM_001278.4
CIC	wholeExon	Y	NM_015125.4

CREBBP	wholeExon	Y	NM_004380.2
CRKL	wholeExon	Y	NM_005207.3
CRLF2	wholeExon	Y	NM_022148.3
CSF1R	wholeExon	Y	NM_001288705.1
CSF3R	selectedExon	Y	NM_156039.3
CTCF	wholeExon	Y	NM_006565.3
CTNNB1	wholeExon	Y	NM_001904.3
CUL3	wholeExon	Y	NM_001257198.1
CUL4A	selectedExon	Y	NM_001008895.2
CUL4B	selectedExon	Y	NM_003588.3
CYLD	selectedExon	Y	NM_015247.2
DAXX	wholeExon	Y	NM_001141970.1
DDR2	wholeExon	Y	NM_001014796.1
DICER1	wholeExon	Y	NM_177438.2
DNMT1	selectedExon	Y	NM_001130823.2
DNMT3A	wholeExon	Y	NM_022552.4
DNMT3B	selectedExon	Y	NM_006892.3
DOT1L	wholeExon	Y	NM_032482.2
EGFR	wholeExon/SNPCNV	Y	NM_005228.3
EIF4E	selectedExon	Y	NM_001130679.1
ELOC	selectedExon	Y	NM_020341.3
EMSY	wholeExon	Y	NM_001300942.1
EP300	wholeExon	Y	NM_001429.3
EPCAM	selectedExon	Y	NM_002354.2
EPHA2	selectedExon	Y	NM_004431.4
EPHA3	wholeExon	Y	NM_005233.5
EPHA5	wholeExon	Y	NM_001281765.2
EPHA7	wholeExon	Y	NM_004440.3
EPHB1	wholeExon	Y	NM_004441.4
ERBB2	wholeExon/SNPCNV	Y	NM_004448.3
ERBB3	wholeExon	Y	NM_001982.3
ERBB4	wholeExon/fusion	Y	NM_005235.2
ERCC1	wholeExon	Y	NM_202001.2
ERCC2	selectedExon	Y	NM_000400.3
ERG	wholeExon	Y	NM_001136154.1
ERRFI1	wholeExon	Y	NM_018948.3
ESR1	wholeExon	Y	NM_000125.3
EWSR1	fusion	Y	NM_013986.3
EZH2	wholeExon	Y	NM_004456.4
FAM175A	wholeExon	Y	NM_139076.2

FANCA	wholeExon	Y	NM_000135.2
FANCC	wholeExon	Y	NM_000136.2
FANCD2	wholeExon	Y	NM_001018115.2
FANCE	wholeExon	Y	NM_021922.2
FANCG	wholeExon	Y	NM_004629.1
FANCI	wholeExon	Y	NM_001113378.1
FANCL	wholeExon	Y	NM_001114636.1
FAT1	wholeExon	Y	NM_005245.3
FAT3	wholeExon	Y	NM_001008781.2
FBXW7	wholeExon	Y	NM_033632.3
FCGR2B	selectedExon	Y	NM_004001.4
FGF12	selectedExon	Y	NM_021032.4
FGF14	selectedExon	Y	NM_175929.2
FGF19	wholeExon/SNPCNV	Y	NM_005117.2
FGF3	wholeExon/SNPCNV	Y	NM_005247.2
FGF4	wholeExon/SNPCNV	Y	NM_002007.2
FGFR1	wholeExon/fusion	Y	NM_023110.2
FGFR2	wholeExon/fusion	Y	NM_000141.4
FGFR3	wholeExon/fusion	Y	NM_000142.4
FGFR4	wholeExon	Y	NM_002011.4
FH	wholeExon	Y	NM_000143.3
FLCN	wholeExon	Y	NM_144997.5
FLT1	wholeExon	Y	NM_002019.4
FLT3	wholeExon	Y	NM_004119.2
FLT4	wholeExon	Y	NM_182925.4
FOXO1	wholeExon	Y	NM_002015.3
FOXP1	wholeExon	Y	NM_001244810.1
FRS2	selectedExon	Y	NM_001042555.2
FUBP1	wholeExon	Y	NM_003902.4
FYN	selectedExon	Y	NM_002037.5
GABRA6	selectedExon	Y	NM_000811.2
GALNT12	wholeExon	Y	NM_024642.4
GATA1	wholeExon	Y	NM_002049.3
GATA2	wholeExon	Y	NM_001145661.1
GATA3	wholeExon	Y	NM_001002295.1
GATA6	selectedExon	Y	NM_005257.5
GLI1	wholeExon	Y	NM_005269.2
GNA11	wholeExon	Y	NM_002067.4
GNAQ	wholeExon	Y	NM_002072.4
GNAS	wholeExon	Y	NM_080425.3

GRIN2A	wholeExon	Y	NM_000833.4
GRM3	wholeExon	Y	NM_000840.2
GSK3B	wholeExon	Y	NM_002093.3
GSTM1	wholeExon	Y	NM_000561.3
GSTT1	wholeExon	Y	NM_000853.3
H3F3A	wholeExon	Y	NM_002107.4
HDAC1	selectedExon	Y	NM_004964.2
HDAC2	selectedExon	Y	NM_001527.3
HDAC4	selectedExon	Y	NM_006037.3
HGF	wholeExon	Y	NM_000601.5
HIST1H3F	selectedExon	Y	NM_021018.2
HLA-A	selectedExon	Y	NM_001242758.1
HNF1A	wholeExon	Y	NM_000545.6
HNF1B	wholeExon	Y	NM_000458.3
HRAS	wholeExon	Y	NM_005343.3
HSP90AA1	selectedExon	Y	NM_001017963.2
IDH1	wholeExon	Y	NM_005896.3
IDH2	wholeExon	Y	NM_001289910.1
IGF1	selectedExon	Y	NM_001111285.2
IGF1R	wholeExon	Y	NM_000875.4
IGF2	wholeExon	Y	NM_000612.5
IKBKE	wholeExon	Y	NM_014002.3
IKZF1	wholeExon	Y	NM_006060.5
IL7R	wholeExon	Y	NM_002185.3
INHBA	wholeExon	Y	NM_002192.3
INPP4A	selectedExon	Y	NM_001134224.1
INPP4B	wholeExon	Y	NM_001101669.1
INSR	selectedExon	Y	NM_000208.3
IRF2	selectedExon	Y	NM_002199.3
IRF4	wholeExon	Y	NM_002460.3
IRS2	wholeExon	Y	NM_003749.2
JAK1	wholeExon	Y	NM_001320923.1
JAK2	wholeExon	Y	NM_004972.3
JAK3	wholeExon	Y	NM_000215.3
KAT6A	selectedExon	Y	NM_006766.4
KDM5A	wholeExon	Y	NM_001042603.2
KDM5C	wholeExon	Y	NM_004187.3
KDM6A	wholeExon	Y	NM_001291415.1
KDR	wholeExon	Y	NM_002253.2
KEAP1	wholeExon	Y	NM_012289.3

KEL	selectedExon	Y	NM_000420.2
KIT	wholeExon	Y	NM_000222.2
KMT2A	wholeExon	Y	NM_001197104.1
KMT2C	wholeExon	Y	NM_170606.2
KMT2D	wholeExon	Y	NM_003482.3
KRAS	wholeExon/SNPCNV	Y	NM_033360.3
LATS1	wholeExon	Y	NM_004690.3
LATS2	selectedExon	Y	NM_014572.2
LMO1	wholeExon	Y	NM_002315.2
LRP1B	wholeExon	Y	NM_018557.2
MAGI2	selectedExon	Y	NM_012301.3
MAP2K1	wholeExon	Y	NM_002755.3
MAP2K2	wholeExon	Y	NM_030662.3
MAP2K4	wholeExon	Y	NM_001281435.1
MAP3K1	wholeExon	Y	NM_005921.1
MAP3K13	selectedExon	Y	NM_001242314.1
MAPK3	selectedExon	Y	NM_002746.2
MAX	selectedExon	Y	NM_002382.4
MCL1	wholeExon	Y	NM_021960.4
MDC1	selectedExon	Y	NM_014641.2
MDM2	wholeExon	Y	NM_002392.5
MDM4	wholeExon	Y	NM_002393.4
MED12	wholeExon	Y	NM_005120.2
MEF2B	wholeExon	Y	NM_001145785.1
MEN1	wholeExon	Y	NM_000244.3
MET	wholeExon/fusion/SNPCNV	Y	NM_000245.3
MITF	wholeExon	Y	NM_000248.3
MLH1	wholeExon	Y	NM_000249.3
MLH3	wholeExon	Y	NM_001040108.1
MPL	wholeExon	Y	NM_005373.2
MRE11A	wholeExon	Y	NM_005591.3
MSH2	wholeExon	Y	NM_000251.2
MSH3	wholeExon	Y	NM_002439.4
MSH6	wholeExon	Y	NM_000179.2
MST1	selectedExon	Y	NM_020998.3
MST1R	selectedExon	Y	NM_002447.3
MTOR	wholeExon	Y	NM_004958.3
MUTYH	wholeExon	Y	NM_001128425.1
MYC	wholeExon	Y	NM_002467.4
MYCL	wholeExon	Y	NM_001033082.2

MYCN	wholeExon	Y	NM_001293228.1
MYD88	wholeExon	Y	NM_001172567.1
NBN	wholeExon	Y	NM_002485.4
NCOR1	selectedExon	Y	NM_006311.3
NEB	selectedExon	Y	NM_001271208.1
NF1	wholeExon	Y	NM_000267.3
NF2	wholeExon	Y	NM_000268.3
NFE2L2	wholeExon	Y	NM_006164.4
NFKBIA	wholeExon	Y	NM_020529.2
NKX2-1	wholeExon	Y	NM_001079668.2
NOTCH1	wholeExon	Y	NM_017617.4
NOTCH2	wholeExon	Y	NM_024408.3
NOTCH3	wholeExon	Y	NM_000435.2
NOTCH4	selectedExon	Y	NM_004557.3
NPM1	wholeExon	Y	NM_002520.6
NR4A3	selectedExon	Y	NM_173200.2
NRAS	wholeExon	Y	NM_002524.4
NRG1	fusion	Y	NM_001322205.1
NSD1	wholeExon	Y	NM_022455.4
NTHL1	wholeExon	Y	NM_002528.6
NTRK1	wholeExon/fusion	Y	NM_001007792.1
NTRK2	wholeExon/fusion	Y	NM_006180.4
NTRK3	wholeExon/fusion	Y	NM_001012338.2
NUP93	wholeExon	Y	NM_014669.4
PAK1	selectedExon	Y	NM_001128620.1
PAK3	selectedExon	Y	NM_001128168.2
PAK7	selectedExon	Y	NM_020341.3
PALB2	wholeExon	Y	NM_024675.3
PARK2	wholeExon	Y	NM_004562.2
PARP2	selectedExon	Y	NM_005484.3
PARP3	selectedExon	Y	NM_001003931.3
PARP4	selectedExon	Y	NM_006437.3
PAX5	wholeExon	Y	NM_016734.2
PBRM1	wholeExon	Y	NM_018313.4
PDGFRA	wholeExon	Y	NM_006206.4
PDGFRB	wholeExon	Y	NM_002609.3
PGR	selectedExon	Y	NM_000926.4
PIK3C2G	selectedExon	Y	NM_001288772.1
PIK3CA	wholeExon	Y	NM_006218.3
PIK3CB	wholeExon	Y	NM_006219.2

PIK3CD	selectedExon	Y	NM_005026.3
PIK3CG	wholeExon	Y	NM_001282426.1
PIK3R1	wholeExon	Y	NM_181523.2
PIK3R2	wholeExon	Y	NM_005027.3
PIM1	selectedExon	Y	NM_001243186.1
PLCG2	wholeExon	Y	NM_002661.4
PMS1	wholeExon	Y	NM_000534.4
PMS2	wholeExon	Y	NM_000535.6
POLD1	wholeExon	Y	NM_001256849.1
POLE	wholeExon	Y	NM_006231.3
PPP2R1A	wholeExon	Y	NM_014225.5
PPP2R2A	wholeExon	Y	NM_001177591.1
PPP6C	selectedExon	Y	NM_001123355.1
PRDM1	wholeExon	Y	NM_001198.3
PREX2	selectedExon	Y	NM_024870.3
PRKAR1A	wholeExon	Y	NM_002734.4
PRKDC	wholeExon	Y	NM_006904.6
PTCH1	wholeExon	Y	NM_000264.3
PTEN	wholeExon	Y	NM_000314.6
PTK2	selectedExon	Y	NM_005607.4
PTPN11	wholeExon	Y	NM_002834.3
PTPRD	wholeExon	Y	NM_002839.3
PTPRS	selectedExon	Y	NM_002850.3
PTPRT	selectedExon	Y	NM_133170.3
QKI	selectedExon	Y	NM_006775.2
RAC1	wholeExon	Y	NM_018890.3
RAD50	wholeExon	Y	NM_005732.3
RAD51	wholeExon	Y	NM_001164269.1
RAD51B	wholeExon	Y	NM_001321821.1
RAD51C	wholeExon	Y	NM_058216.2
RAD51D	wholeExon	Y	NM_002878.3
RAD52	wholeExon	Y	NM_001297419.1
RAD54L	wholeExon	Y	NM_001142548.1
RAF1	wholeExon	Y	NM_002880.3
RARA	wholeExon	Y	NM_000964.3
RASA1	selectedExon	Y	NM_002890.2
RB1	wholeExon	Y	NM_000321.2
RBM10	wholeExon	Y	NM_001204468.1
RECQL4	selectedExon	Y	NM_004260.3
RET	wholeExon/fusion	Y	NM_020975.4

RHOA	selectedExon	Y	NM_001664.3
RICTOR	wholeExon	Y	NM_001285439.1
RNF43	wholeExon	Y	NM_017763.5
ROS1	wholeExon/fusion	Y	NM_002944.2
RPS6KA4	selectedExon	Y	NM_003942.2
RPS6KB2	selectedExon	Y	NM_003952.2
RPTOR	wholeExon	Y	NM_020761.2
RUNX1	wholeExon	Y	NM_001754.4
RUNX1T1	selectedExon	Y	NM_001198679.1
SDHA	wholeExon	Y	NM_004168.3
SDHB	wholeExon	Y	NM_003000.2
SDHC	wholeExon	Y	NM_003001.3
SDHD	wholeExon	Y	NM_003002.3
SETD2	wholeExon	Y	NM_014159.6
SF3B1	wholeExon	Y	NM_001005526.2
SH2B3	selectedExon	Y	NM_005475.2
SLIT2	selectedExon	Y	NM_004787.3
SLX4	wholeExon	Y	NM_032444.2
SMAD2	wholeExon	Y	NM_001003652.3
SMAD3	wholeExon	Y	NM_005902.3
SMAD4	wholeExon	Y	NM_005359.5
SMARCA4	wholeExon	Y	NM_001128849.1
SMARCB1	wholeExon	Y	NM_003073.4
SMARCD1	selectedExon	Y	NM_003076.4
SMO	wholeExon	Y	NM_005631.4
SNCAIP	selectedExon	Y	NM_001308100.1
SOX9	wholeExon	Y	NM_000346.3
SPEN	wholeExon	Y	NM_015001.2
SPOP	wholeExon	Y	NM_001007226.1
SPTA1	wholeExon	Y	NM_003126.2
SRC	wholeExon	Y	NM_198291.2
SRSF2	wholeExon	Y	NM_003016.4
STAG2	wholeExon	Y	NM_001042749.2
STAT3	wholeExon	Y	NM_139276.2
STAT5A	selectedExon	Y	NM_001288718.1
STAT5B	wholeExon	Y	NM_012448.3
STK11	wholeExon	Y	NM_000455.4
STK40	selectedExon	Y	NM_001282546.1
SUFU	wholeExon	Y	NM_016169.3
SYK	wholeExon	Y	NM_001174167.2

TAF1	selectedExon	Y	NM_001286074.1
TBX3	wholeExon	Y	NM_016569.3
TCF3	selectedExon	Y	NM_003200.3
TCF7L2	selectedExon	Y	NM_001146274.1
TERT	wholeExon/Promoter	Y	NM_198253.2
TET2	wholeExon	Y	NM_001127208.2
TGFBR2	wholeExon	Y	NM_001024847.2
TMPRSS2	fusion	Y	NM_001135099.1
TNFAIP3	wholeExon	Y	NM_001270507.1
TNFRSF14	wholeExon	Y	NM_003820.3
TNFSF11	wholeExon	Y	NM_003701.3
TOP1	wholeExon	Y	NM_003286.2
TP53	wholeExon	Y	NM_000546.5
TP63	wholeExon	Y	NM_003722.4
TRAF2	selectedExon	Y	NM_021138.3
TRAF7	selectedExon	Y	NM_032271.2
TRRAP	selectedExon	Y	NM_001244580.1
TSC1	wholeExon	Y	NM_000368.4
TSC2	wholeExon	Y	NM_000548.4
TSHR	wholeExon	Y	NM_000369.2
U2AF1	wholeExon	Y	NM_001025203.1
VEGFA	wholeExon	Y	NM_001025366.2
VHL	wholeExon	Y	NM_000551.3
WRN	wholeExon	Y	NM_000553.4
WT1	wholeExon	Y	NM_024426.4
XPO1	wholeExon	Y	NM_003400.3
XRCC2	wholeExon	Y	NM_005431.1
YES1	selectedExon	Y	NM_005433.3
ZFHX3	selectedExon	Y	NM_006885.3
ZNF217	selectedExon	Y	NM_006526.2
ZNF703	selectedExon	Y	NM_025069.2
ZNRF3	selectedExon	Y	NM_001206998.1
AMER1	wholeExon	N	NM_152424.3
APCDD1	selectedExon	N	NM_153000.4
ARFRP1	selectedExon	N	NM_001267547.2
BACH1	selectedExon	N	NM_001186.3
BBC3	selectedExon	N	NM_001127240.2
BCL10	selectedExon	N	NM_003921.4
BCL2L11	Intron	N	NM_001204107.1
BCL2L2	selectedExon	N	NM_001199839.1

BTG1	selectedExon	N	NM_001731.2
CALR	selectedExon	N	NM_004343.3
CD276	selectedExon	N	NM_001024736.1
CENPA	selectedExon	N	NM_001809.3
CRBN	selectedExon	N	NM_016302.3
CTLA4	selectedExon	N	NM_005214.4
CTNNA1	selectedExon	N	NM_001323982.1
CXCR4	selectedExon	N	NM_003467.2
CYP17A1	selectedExon	N	NM_000102.3
DCUN1D1	selectedExon	N	NM_020640.3
DIS3	selectedExon	N	NM_014953.4
DNAJB1	selectedExon	N	NM_006145.2
E2F3	selectedExon	N	NM_001949.4
EED	selectedExon	N	NM_001308007.1
EGFL7	selectedExon	N	NM_016215.4
EIF1AX	selectedExon	N	NM_001412.3
EIF4A2	selectedExon	N	NM_001967.3
ERCC3	selectedExon	N	NM_000122.1
ERCC4	selectedExon	N	NM_005236.2
ERCC5	selectedExon	N	NM_000123.3
FAM46C	wholeExon	N	NM_017709.3
FANCF	wholeExon	N	NM_022725.3
FANCM	selectedExon	N	NM_020937.3
FAS	selectedExon	N	NM_000043.5
FGF10	selectedExon	N	NM_004465.1
FGF23	selectedExon	N	NM_020638.2
FGF6	selectedExon	N	NM_020996.2
FGF7	selectedExon	N	NM_002009.3
FOXA1	selectedExon	N	NM_004496.3
FOXL2	wholeExon	N	NM_023067.3
GATA4	selectedExon	N	NM_001308093.1
GID4	selectedExon	N	NM_024052.4
GNA13	selectedExon	N	NM_006572.5
GPS2	selectedExon	N	NM_004489.4
GREM1	wholeExon	N	NM_013372.6
H3F3B	selectedExon	N	NM_005324.4
H3F3C	wholeExon	N	NM_001013699.2
HIST1H1C	wholeExon	N	NM_005319.3
HIST1H2BD	wholeExon	N	NM_021063.3
HIST1H3A	wholeExon	N	NM_003529.2

HIST1H3B	wholeExon	N	NM_003537.3
HIST1H3C	wholeExon	N	NM_003531.2
HIST1H3D	wholeExon	N	NM_003530.4
HIST1H3E	wholeExon	N	NM_003532.2
HIST1H3G	wholeExon	N	NM_003534.2
HIST1H3H	wholeExon	N	NM_003536.2
HIST1H3I	wholeExon	N	NM_003533.2
HIST1H3J	wholeExon	N	NM_003535.2
HIST2H3C	selectedExon	N	NM_021059.2
HIST2H3D	wholeExon	N	NM_001123375.2
HIST3H3	wholeExon	N	NM_003493.2
HOXB13	selectedExon	N	NM_006361.5
HSD3B1	selectedExon	N	NM_000862.2
ICOSLG	selectedExon	N	NM_001283050.1
ID3	selectedExon	N	NM_002167.4
IFNGR1	selectedExon	N	NM_000416.2
IL10	selectedExon	N	NM_000572.2
INHA	selectedExon	N	NM_002191.3
IRS1	wholeExon	N	NM_005544.2
JUN	wholeExon	N	NM_002228.3
KLF4	selectedExon	N	NM_001314052.1
KLHL6	selectedExon	N	NM_130446.2
LYN	selectedExon	N	NM_002350.3
LZTR1	selectedExon	N	NM_006767.3
MALT1	selectedExon	N	NM_006785.3
MAP3K14	selectedExon	N	NM_003954.4
MAPK1	selectedExon	N	NM_002745.4
MGA	selectedExon	N	NM_001164273.1
MYOD1	selectedExon	N	NM_002478.4
NCOA3	selectedExon	N	NM_181659.2
NEGR1	selectedExon	N	NM_173808.2
NKX3-1	selectedExon	N	NM_006167.3
PARP1	selectedExon	N	NM_001618.3
PDCD1	selectedExon	N	NM_005018.2
PDCD1LG2	selectedExon	N	NM_025239.3
PDK1	selectedExon	N	NM_001278549.1
PDPK1	selectedExon	N	NM_002613.4
PHOX2B	selectedExon	N	NM_003924.3
PIK3C2B	selectedExon	N	NM_002646.3
PIK3C3	selectedExon	N	NM_002647.3

PIK3R3	selectedExon	N	NM_001303427.1
PLK2	selectedExon	N	NM_006622.3
PMAIP1	selectedExon	N	NM_021127.2
PNRC1	selectedExon	N	NM_006813.2
POM121L12	wholeExon	N	NM_182595.3
PPM1D	selectedExon	N	NM_003620.3
PRKCI	selectedExon	N	NM_002740.5
PRSS8	selectedExon	N	NM_002773.4
RAB35	selectedExon	N	NM_006861.6
RAD21	selectedExon	N	NM_006265.2
RANBP2	selectedExon	N	NM_006267.4
REL	selectedExon	N	NM_002908.3
RFWD2	selectedExon	N	NM_022457.6
RHEB	selectedExon	N	NM_005614.3
RIT1	selectedExon	N	NM_001256821.1
RPA1	selectedExon	N	NM_002945.3
RYBP	selectedExon	N	NM_012234.6
SDHAF2	selectedExon	N	NM_017841.2
SH2D1A	selectedExon	N	NM_002351.4
SHQ1	selectedExon	N	NM_018130.2
SOCS1	wholeExon	N	NM_003745.1
SOX10	selectedExon	N	NM_006941.3
SOX17	selectedExon	N	NM_022454.3
SOX2	wholeExon	N	NM_003106.3
STAT4	selectedExon	N	NM_001243835.1
SUZ12	selectedExon	N	NM_015355.3
TACC3	selectedExon	N	NM_006342.2
TERC	wholeExon	N	NR_001566.1
TET1	selectedExon	N	NM_030625.2
TGFBR1	selectedExon	N	NM_001306210.1
TIPARP	selectedExon	N	NM_001184717.1
TMEM127	selectedExon	N	NM_017849.3
TOP2A	selectedExon	N	NM_001067.3
VEGFB	selectedExon	N	NM_003377.4
VEGFC	selectedExon	N	NM_005429.4
VTCN1	selectedExon	N	NM_024626.3
WISP3	selectedExon	N	NM_198239.1
XIAP	selectedExon	N	NM_001167.3
XRCC3	selectedExon	N	NM_001100118.1
YAP1	selectedExon	N	NM_001282101.1

ZBTB2	selectedExon	N	NM_020861.2
ZRSR2	selectedExon	N	NM_005089.3
