# Supplementary material for: Somatic POLE exonuclease domain mutations elicit enhanced intratumoral immune responses in stage II colorectal cancer
Source: J Immunother Cancer. 2020 Aug 27;8(2):e000881. doi: 10.1136/jitc-2020-000881 (PMC7454238; doi:10.1136/jitc-2020-000881)
Supplement: Supplementary data [file jitc-2020-000881supp014.pdf]

Supplementary Table 8. Immunohistochemical multiplex staining protocol.

Panel 1

No.	1	2	3	4	5
Antigen	**CD45RO**	**PDL1**	**CD8**	**CD3**	**PD1**
Catalogue number	ZM-0055	ZA-0629	ZA0508	ZM-0417	ZM0381
Species	mouse	Rabbit	Rabbit	Mouse	Mouse
Concentration	1:200	1:25	1:100	1:50	1:100
Antigen retrieval	AR9	AR9	AR9	AR9	AR9
Incubation	37℃ 1h	37℃ 1Hr	4℃ ON	37℃ 1Hr	37℃ 1Hr
Secondary antibody	PV-9000	PV-8000	PV-9000	PV-9000	PV-8000
TSA	620	570	520	690	650

Panel 2

No.	1	2	3	4	5
Antigen	**FoxP3**	**CD163**	**CD68**	**CD4**	**PDL1**
Catalogue number	ab20034	ZM0428	ZM0060	ZM0418	ZA0629
Species	Mouse	Mouse	Mouse	Mouse	Rabbit
Concentration	1:400	1:100	1:500	Working solution	1:25
Antigen retrieval	AR9	AR9	AR9	AR9	AR9
Incubation	37℃ 1Hr	37℃ 1Hr	4℃ ON	37℃ 1Hr	37℃ 1Hr
Secondary antibody	PV-9000	PV-8000	PV-9000	PV-8000	PV-8000
TSA	650	520	690	570	540
