# Supplementary material for: Somatic POLE exonuclease domain mutations elicit enhanced intratumoral immune responses in stage II colorectal cancer
Source: J Immunother Cancer. 2020 Aug 27;8(2):e000881. doi: 10.1136/jitc-2020-000881 (PMC7454238; doi:10.1136/jitc-2020-000881)
Supplement: Supplementary data [file jitc-2020-000881supp007.pdf]

Supplementary Table 1. Demographic and clinicopathological characteristics according to stage II CRC POLE status in TCGA cohort.

Characteristic	POLE EDMs	POLE wild-type & POLE non-EDMs	p value
Total	7 (3.8)	176 (96.2)	--
Age (IQR)	59 (46-70)	69 (60-78)	0.049
Year of diagnosis			0.578
1998-2005	1 (14.3)	41 (23.3)	
2006-2013	6 (85.7)	135 (76.7)	
Gender			0.305
Female	2 (28.6)	85 (48.3)	
Male	5 (71.4)	91 (51.7)	
Histological type			0.946
Adenocarcinoma	6 (85.7)	153 (86.9)	
Mucinous adenocarcinoma	1 (14.3)	21 (11.9)	
Unknown	0 (0)	2 (1.2)	
Location			0.283
Right	3 (42.8)	74 (42.0)	
Left	2 (28.6)	85 (48.3)	
Unknown	2 (28.6)	17 (9.7)	
pT stage			0.024
T3	5 (71.4)	165 (93.8)	
T4	2 (28.6)	11 (6.2)	
Venous invasion			0.979
Negative	5 (71.4)	120 (68.2)	
Positive	1 (14.3)	30 (17.0)	
Unknown	1 (14.3)	26 (14.8)	
Lymphatic invasion			0.808
Negative	4 (57.1)	119 (67.6)	
Positive	2 (28.6)	42 (23.9)	
Unknown	1 (14.3)	15 (8.5)	
Perineural invasion			0.631
Negative	2 (28.6)	55 (31.3)	
Positive	0 (0)	18 (10.2)	
Unknown	5 (71.4)	103 (58.5)	
LNH			0.885
< 12	1 (14.3)	20 (11.4)	
≥ 12	5 (71.4)	139 (79.0)	
Unknown	1 (14.3)	17 (9.6)	
MSI status			0.355
MSS	7 (100)	134 (76.1)	
MSI-H	0 (0)	42 (23.9)	

CRC, colorectal cancer; EDM, exonuclease domain mutation; IQR, interquartile range; LNH,

number of lymph nodes harvested; MSI, microsatellite instability; MSS, microsatellite stabilization
